# GITR and TIGIT immunotherapy provokes divergent multicellular responses in the tumor microenvironment of gastrointestinal cancers

**DOI:** 10.1186/s13073-023-01259-3

**Published:** 2023-11-26

**Authors:** Anuja Sathe, Carlos Ayala, Xiangqi Bai, Susan M. Grimes, Byrne Lee, Cindy Kin, Andrew Shelton, George Poultsides, Hanlee P. Ji

**Affiliations:** 1 Division of Oncology, Department of Medicine, Stanford University School of Medicine, CCSR 2245, 269 Campus Drive, Stanford, CA 94305 USA; 2 Division of Surgical Oncology, Department of Surgery, Stanford University, Stanford, CA USA

**Keywords:** GITR, TIGIT, Tumor microenvironment, scRNA-seq, Gastric cancer, Colon cancer

## Abstract

**Background:**

Understanding the mechanistic effects of novel immunotherapy agents is critical to improving their successful clinical translation. These effects need to be studied in preclinical models that maintain the heterogenous tumor microenvironment (TME) and dysfunctional cell states found in a patient’s tumor. We investigated immunotherapy perturbations targeting co-stimulatory molecule GITR and co-inhibitory immune checkpoint TIGIT in a patient-derived ex vivo system that maintains the TME in its near-native state. Leveraging single-cell genomics, we identified cell type-specific transcriptional reprogramming in response to immunotherapy perturbations.

**Methods:**

We generated ex vivo tumor slice cultures from fresh surgical resections of gastric and colon cancer and treated them with GITR agonist or TIGIT antagonist antibodies. We applied paired single-cell RNA and TCR sequencing to the original surgical resections, control, and treated ex vivo tumor slice cultures. We additionally confirmed target expression using multiplex immunofluorescence and validated our findings with RNA in situ hybridization.

**Results:**

We confirmed that tumor slice cultures maintained the cell types, transcriptional cell states and proportions of the original surgical resection. The GITR agonist was limited to increasing effector gene expression only in cytotoxic CD8 T cells. Dysfunctional exhausted CD8 T cells did not respond to GITR agonist. In contrast, the TIGIT antagonist increased TCR signaling and activated both cytotoxic and dysfunctional CD8 T cells. This included cells corresponding to TCR clonotypes with features indicative of potential tumor antigen reactivity. The TIGIT antagonist also activated T follicular helper-like cells and dendritic cells, and reduced markers of immunosuppression in regulatory T cells.

**Conclusions:**

We identified novel cellular mechanisms of action of GITR and TIGIT immunotherapy in the patients’ TME. Unlike the GITR agonist that generated a limited transcriptional response, TIGIT antagonist orchestrated a multicellular response involving CD8 T cells, T follicular helper-like cells, dendritic cells, and regulatory T cells. Our experimental strategy combining single-cell genomics with preclinical models can successfully identify mechanisms of action of novel immunotherapy agents. Understanding the cellular and transcriptional mechanisms of response or resistance will aid in prioritization of targets and their clinical translation.

**Supplementary Information:**

The online version contains supplementary material available at 10.1186/s13073-023-01259-3.

## Background

The success of checkpoint blockade for cancer immunotherapy has spurred on the development of new immune-related therapeutic targets. However, our knowledge of the mechanism of action of these agents is still limited. To understand the mechanistic effects of these immunotherapy agents in cancer, one must evaluate their impact on the diverse cell types that are present within the tumor microenvironment (TME). Cell culture systems of T cell exhaustion and mouse cancer models are commonly used to evaluate immunotherapy agents. However, neither of these experimental approaches replicate the cellular diversity found in the native TME within patients’ malignancies [1].

In patient tumors, the native TME has a wide array of cell types including T cells, fibroblasts, and macrophages. Moreover, many cell types within the native TME have specific functional phenotypes that are a challenge to replicate within in vitro systems. For example, TME-based CD8 T cells exhibit naïve, cytotoxic, or exhausted functional phenotypes [2]. These cellular functional “states” have a profound impact on response to an immuno-perturbation. Thus, there are significant advantages for using experimental methods that fully represent the TME cellular complexity, identify the different cell types, and determine their functional states. A recent study assessed the cellular effects of PD-1 blockade on ex vivo fragment cultures of patient tumor specimens [3]. Early ex vivo cytokine and chemokine responses at 48 h correlated with clinical response. Thus, using primary tissue cultures has utility for evaluating cellular responses in the TME to understand immunotherapy effects.

A method for preserving cellular composition of cancers involves tumor slice cultures (TSCs) [4]. Tumors originating from surgical resections are rapidly processed into thin slices and then placed in culture media. The tissue sections’ thickness is in the range of several hundred microns which enables rapid diffusion of media, oxygen and other molecules. This primary tissue culture approach has been well-established in preserving the cellular TME of the original tumor [4–7]. While TSCs have been used to evaluate the effects of chemotherapy agents in primary tumor specimens [8–10], only a limited number of recent studies have leveraged them to determine the consequences of immunotherapies such as anti-PD-1, anti-TIM-3, anti-IL-10 and CAR-T cells [11, 12].

There are additional challenges for evaluating the impact of targeting specific immune blockade molecules. Conventional experimental methods do not provide the resolution to identify the complex features of individual TME cells. Many studies use fluorescent antibody staining approaches to identify specific cells, either through flow cytometry or microscopy. However, these methods capture a limited number of pre-defined molecular features among the affected cells. Another experimental approach involves using conventional RNA-seq to identify gene expression changes in the TME. However, standard RNA-seq requires processing the tissues in bulk and lacks the discrimination of assigning gene expression to individual cell types present in the TME. More recently, single-cell RNA sequencing (scRNA-seq) has provided an unbiased assessment of individual cell’s transcriptional changes. Single-cell gene expression defines specific cell types or functional states. These single-cell methods have provided valuable information about how specific cell types respond to PD-1 blockade using longitudinal pre- and on-treatment patient biopsies [13, 14].

To address the challenges of studying the effects of candidate immunotherapies in the native TME, we used an integrative approach, combining the TSC experimental model with single-cell genomics. We determined how specific antibodies targeting immune checkpoints or costimulatory molecules altered the immune and other cell types present in the native TME from gastrointestinal cancers. To evaluate the cellular effects, we used scRNA-seq and single-cell TCR sequencing (scTCR-seq). Across a series of TSCs derived from colorectal and gastric carcinomas, we determined the cellular response of specific immune perturbations, namely antibodies targeting specific checkpoints or costimulatory molecules. Single-cell gene expression provided a readout to determine how specific TME cell subpopulations were affected by these perturbations.

We tested antibodies against the timmunotherapy targets GITR and TIGIT, respectively. Antibodies targeting GITR and TIGIT are both being actively evaluated in various clinical trials for cancer [15]. GITR is a co-stimulatory T cell receptor [16]. TIGIT is a co-inhibitory receptor, which binds with ligands from the PVR/NECTIN family and reduces the costimulatory function of the CD226 receptor [17]. Previously, we identified both these targets in a single-cell genomic analysis of gastric cancers’ (GC) TME [18]. These targets were over-expressed in both exhausted CD8 T cells and regulatory T cells (Tregs) in the TME but not in paired normal gastric tissue. Similar findings have been reported in colorectal cancer (CRC) [19] and several solid tumors [20]. As part of this study, we confirmed GITR and TIGIT protein expression in a series of colorectal and gastric cancers and confirmed the expression of *GITR* and *TIGIT* from independent data sets [21].

We determined that GITR agonist antibody had only a limited cellular effect which was primarily restricted to cytotoxic effector CD8 T cells. In contrast, when we tested a TIGIT antagonistic antibody on TSCs from the same set of cancers, we observed increased TCR signaling and activation in both cytotoxic and dysfunctional CD8 T cells, including in expanded clonotypes. Moreover, using the TIGIT antagonist antibody, we observed activated follicular helper-like (TFh-like) cells and a reduction in the immunosuppressive phenotype of Tregs and dendritic cells (DCs). These results demonstrated how single-cell genomics combined with TSCs can be applied to primary gastrointestinal cancers to identify the heterogenous cellular responses to GITR stimulation and TIGIT inhibition.

## Methods

### Samples

We obtained informed consent from all patients based on a protocol approved by the Stanford University Institutional Review Board. All samples were surgical tumor resections obtained from patients undergoing surgery at the Department of Surgery, Stanford University. They included three gastric cancer resections obtained from one individual patient and seven colorectal cancer resections from seven individual patients. Clinical pathology report that was generated at the time of the resection was reviewed for all samples. Single-cell sequencing was performed on baseline surgical resections (“T0”) from all ten resections. Tissue slice cultures were generated from all resections and subjected to perturbations before undergoing single-cell sequencing. Perturbations included control (*n*=10), PMA/Ionomycin (*n*=8), GITR agonist (*n*=9), and TIGIT antagonist (*n*=5).

### Tissue processing

Tissues were collected in plain RPMI on ice immediately after resection and dissected with iris scissors. From the original T0 surgical resections, a portion was fixed for histopathology, a portion was subjected to dissociation, and the remainder was used to generate tumor slice cultures.

### Ex vivo tumor slice cultures (TSCs)

A VF-310-0Z Compresstome tissue slicer and its accessories (Precisionary, Greenville, NC, USA) were used to generate tissue slices from a piece of the resection. Tissue sample was glued onto the specimen tube base using All Purpose Krazy Glue (Elmer’s Products, Inc., Westerville, OH, USA). The 3% agarose solution was prepared by diluting UltraPure Low Melting Point Agarose (Thermo Fisher Scientific) in water followed by heating in a microwave and cooling for around 3 min at room temperature. The tissue sample was retracted into the specimen tube and covered with agarose solution. Agarose was solidified by placing pre-chilled chilling block supplied by the manufacturer over the specimen tube. Specimen tube was assembled onto compresstome as per the manufacturer’s instructions and cold PBS was used as a solution in the buffer tank. Slices were generated using advance setting of 3, oscillation of 5, and thickness of 400 μm. Slices were placed onto a 0.4 μm pore size Millicell Cell Culture Insert (Sigma-Aldrich, St. Louis, MO, USA) that was then placed into a 35-mm dish (Thermo Fisher Scientific). The media volume included 1.5 ml that was placed into the surrounding dish and 0.5 ml placed onto the slices followed by culture in a cell culture incubator. Media was composed of RPMI, 10% FBS, and 1% Antibiotic-Antimycotic (Thermo Fisher Scientific). Perturbations were added to the media once at the beginning of culture. Two micrograms/milliliter IgG1 Fc (BPS Bioscience, catalog #71456) was used as control. Treatment conditions included 2 μg/ml GITR agonist (BPS Bioscience, catalog #79053), 2 μg/ml TIGIT antagonist (BPS Bioscience, catalog #71340), or 6 μg/ml eBioscience Cell Stimulation Cocktail (500X) (Thermo Fisher Scientific). At 24 h, TSCs were subjected to fixation for histology and dissociation.

### Histopathology

Tissue was fixed in 10% formalin for approximately 24 h at room temperature. Paraffin embedding and hematoxylin and eosin staining was conducted by the Human Pathology Histology Services core facility at Stanford University. Whole slide images were obtained using Aperio AT2 whole slide scanner (Leica Biosystems Inc., IL, USA). Tissue fixation was not performed for T0 sample CRC-3 due to inadequate material.

### Single-cell dissociation

Tissue dissociation was conducted using a combination of enzymatic and mechanical dissociation using a gentleMACS Octo Dissociator (Miltenyi Biotec) as described previously [18]. Cells were cryofrozen using 10% DMSO in 90% FBS (Thermo Fisher Scientific, Waltham, MA) in a CoolCell freezing container (Larkspur, CA) at −80 °C for 24–72 h followed by storage in liquid nitrogen. For scRNA-seq, cryofrozen cells were rapidly thawed in a bead bath at 37 °C, washed twice in RPMI + 10% FBS, and filtered successively through 70- and 40-μm filters (Flowmi, Bel-Art SP Scienceware, Wayne, NJ). Live cell counts were obtained using 1:1 trypan blue dilution. Cells were concentrated between 500-1500 live cells/μl.

### Single-cell RNA sequencing

The scRNA-seq libraries were generated from cell suspensions using Chromium Next GEM Single Cell 5’ version 1.1 (samples CRC-1, CRC-2, GC1-1, GC-1-2, GC-1-3) or version 2 (samples CRC-3, CRC-4, CRC-5, CRC-6, CRC-7) (10X Genomics, Pleasanton, CA, USA) as per the manufacturer’s protocol. All libraries from a patient were prepared in the same experimental batch. Ten thousand cells were targeted with 14 PCR cycles for cDNA and library amplification. Chromium Single Cell V(D)J Human T Cell Enrichment Kit was used to prepare TCR libraries from single-cell cDNA as per the manufacturer’s protocol. A 1 or 2% E-Gel (Thermo Fisher Scientific, Waltham, MA, USA) was used for quality control evaluation of intermediate products and sequencing libraries. Qubit (Thermo Fisher Scientific) was used to quantify the libraries as per the manufacturer’s protocol. Libraries were sequenced on Illumina sequencers (Illumina, San Diego, CA).

### Data processing of scRNA-seq

Cell Ranger (10x Genomics) version 3.1.0 or 5.0.0 “mkfastq” command was used for NextGEM version 1.1 and version 2 libraries respectively to generate Fastq files. Cell Ranger version 3.1.0 “count” was used with default parameters and alignment to GRCh38 to generate a matrix of unique molecular identifier (UMI) counts per gene and associated cell barcode. Cell Ranger version 6.0.0 “vdj” command was used to perform sequence assembly and clonotype calling of TCR libraries with alignment to the prebuilt Cell Ranger V(D)J reference version 5.0.0 for GRCh38.

### Clustering individual datasets

We constructed Seurat objects from each sample using Seurat (version 4.0.1) [22, 23]. We applied quality control filters to remove cells that expressed fewer than 200 genes, had greater than 30% mitochondrial genes, or had UMI counts greater than 8000 as an indicator of cell doublets. We removed genes that were detected in less than 3 cells. We normalized data using “SCTransform” and used first 20 principal components with a resolution of 0.8 for clustering. We then removed computationally identified doublets from each dataset using DoubletFinder (version 2.0.3) [24]. The “pN” value was set to default value of 0.25 as the proportion of artificial doublets. The “nExP” was set to expected doublet rate according to Chromium Single Cell 3’ version 2 reagents kit user guide (10X Genomics). These parameters were used as input to the “doubletFinder_v3” function with number of principal components set to 20 to identify doublet cells.

### Batch-corrected integrated scRNA-seq analysis

Individual Seurat objects were merged and normalized using “SCTransform” [22, 23]. To eliminate potential batch effects, we integrated all datasets using the Harmony algorithm (version 0.1.0) [25] using patient as the grouping variable in the “RunHarmony” function. Harmony reduction was used in both “RunUMAP” and “FindNeighbors” functions for clustering. The first 20 principal components and a resolution of 2 was used for clustering. The data from the “RNA” assay was used for all further downstream analysis with other packages, gene level visualization, or differential expression analysis. The data was normalized to the logarithmic scale and the effects of variation in sequencing depth were regressed out by including “nCount_RNA” as a parameter in the “ScaleData” function. One integrated object comprised all T0 samples. A second integrated object included all ctrl and treated ex vivo samples.

### Cell lineage identification and reclustering of integrated scRNA-seq data

From both the batch-corrected integrated Seurat objects, cell lineages were identified based on marker gene expression. Clusters lacking marker genes but with high expression of mitochondrial or heat shock protein family genes, and those expressing markers of more than one lineage indicative of doublets were filtered from the downstream analysis [20]. We performed a secondary clustering analysis of each lineage with integration across patients using Harmony and a cluster resolution of 1. Any clusters identified as belonging to another cell lineage were united with their lineage counterparts for a second clustering run. This yielded final lineage-specific reclustering results. In integrated analysis of T0 samples, a single proliferative cluster comprising 2.1% total cells with both B and T cells was gated for T cells based on the expression of normalized counts for *CD3D* or *CD3E* > 0.

**Fig. 1:**
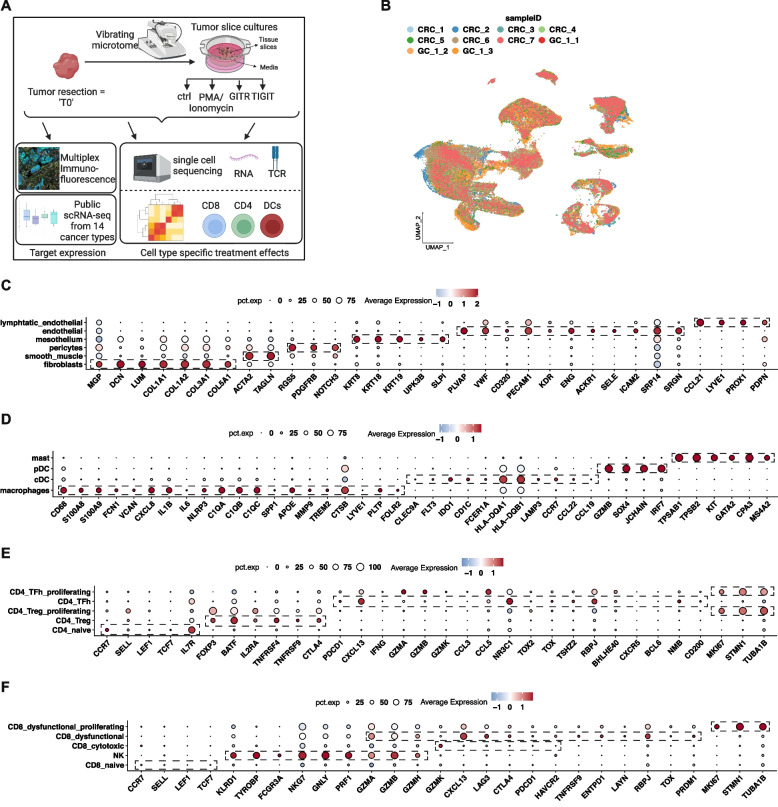
**A** Schematic representation of study design. **B** UMAP representation of dimensionally reduced data following batch-corrected graph-based clustering of all datasets colored by samples. **C**–**F** Dot plot depicting average expression levels of specific lineage-based marker genes together with the percentage of cells expressing the marker in **C** stromal, **D** myeloid, and **E**, **F** lymphocytes

Clusters containing T and NK cells were subjected to further cell type identification. To ensure elimination of B-T doublets, we filtered cells expressing immunoglobulin genes as described previously [20]. Immunoglobulin gene expression was quantified using Seurat “AddModuleScore” function and the cells with expression score >0 were filtered. T and NK cell lineages were identified using a combination of automated and manual annotation as recommended by current best practices guidelines [26, 27]. We used the single-cell tumor-infiltrating immune cell atlas as a reference for automated annotation [21]. Seurat object and metadata for the atlas was obtained from [28] and filtered for cells belonging to T and NK cell lineages. Counts from the reference atlas were normalized to the logarithmic scale and used as a reference for automated annotation per cell using SingleR (version 1.14.1) [29]. Raw counts were used to annotate test datasets. Labels were predicted for each cell in the test dataset using the “SingleR” function to calculate the Spearman correlation for 50 marker genes for the reference dataset identified with Wilcoxon rank sum test. Following automated label assignment using this method, we then confirmed results by examining marker gene expression (Fig. 1E, F) [2, 20, 21]. Cell labels were reannotated or reassigned in case of misassignment in keeping with current recommended best practices [26]. No cells mapped to the “recently activated CD4 T cells” label. T helper cells were renamed as TFh-like cells [2]. Cytotoxic and effector memory CD8 shared marker gene expression and were renamed as cytotoxic CD8 T cells. Pre-exhausted cells were regrouped with terminally exhausted cells based on shared marker gene expression and renamed as dysfunctional CD8 T cells [2]. Naive-memory CD4 T cells were regrouped together with naïve cells based on shared marker gene expression. We classified annotated naïve cells into CD4 or CD8 naïve cells based on marker gene expression. Transitional memory cells expressed marker genes of TFh-like cells and were reassigned. Th17 expressed cytotoxic effector CD8 T cell markers and were reassigned. We identified any dysfunctional CD8 cells misidentified as TFh-like cells based on normalized expression of *CD8A* or *CD8B* > 0. Using marker gene expression, we determined that proliferating cells contained a mix of dysfunctional CD8 T cells, TFh-like cells, and Tregs. These subpopulations were identified using gating for lineage-specific counts. Dysfunctional CD8 T cells were classified based on expression of *CD8A* or *CD8B* > 0 followed by Tregs with *FOXP3* >0 with the remainder as TFh-like cells. A final round of harmonized clustering was performed on control and individual treatment comparisons for lineage of interest, for example, CD8 T cells from ctrl and TIGIT conditions. Following this cell type identification from integrated objects, T0 and ctrl cells were clustered together with batch correction to compare baseline tumor composition with ex vivo models.

### Differential expression

Differential expression analysis between control and treated cells was conducted using model-based analysis of single-cell transcriptomics (MAST) [30] (version 1.18.0) on genes expressed in greater than 10% cells using log normalized data. The number of detected genes were recalculated after filtering. The MAST hurdle model was modelled for the treatment condition and adjusted for the number of detected genes. To account for inter-sample variability across different patients, we incorporated sample as a random effect in the linear mixed model [31]. This was implemented using the “zlm” function in MAST with “glmer” as the method and “ebayes” set to false. A likelihood ratio test was performed on the model coefficients [30, 31]. Expected log fold change was computed between the model coefficients. A threshold of log_2_ fold change of 0.4 and false discovery rate (FDR) adjusted *p* < 0.05 was used to identify significant DE genes. In cases where we examined differences between groups of cells without modelling interpatient variability, we used the “FindAllMarkers” or “FindMarkers” Seurat functions for differential expression using Wilcoxon rank sum test. These instances are identified in the manuscript text. Parameters provided for these functions were as follows: genes detected in at least 25% cells and differential expression threshold of 0.25 ln fold change. Significant genes were determined with *p* < 0.05 following Bonferroni correction.

### Pathway analysis

Gene sets of interest were obtained from MSigDB Human Collections [32] using package msigdbr (version 7.5.1). These included “BIOCARTA_NFKB_PATHWAY” and “REACTOME_TCR_SIGNALING” from curated gene sets, “GO_CELLULAR_RESPONSE_TO_CALCIUM_ION” and “GOBP_T_CELL_ACTIVATION” from biological process gene ontology, “HALLMARK_INTERFERON_GAMMA_RESPONSE” from Hallmark gene sets. Additional gene sets for cytotoxic effector gene signature [33, 34] and CD8 dysfunction [35] were compiled from literature (Additional file 1: Table S5). We used the “AddModuleScore” function in Seurat to calculate the expression of a gene set of interest in each cell using default parameters. Expression between categories was compared using unpaired *t*-test. Effect size was estimated using Cohen’s *d* measure with equal variance and Hedge’s correction implemented in rstatix (version 0.7.0).

### TCR analysis

Filtered Cell Ranger V(D)J outputs indicative of productive TCR chains detected in high-confidence cells were used. In cases of multiple TRA or TRB sequences detected per cell, we retained the sequence with higher UMI count as described previously [20]. In rare instances with ties for UMI count, we retained both sequences per cell. Expansion index in cell types using Shannon entropy was calculated with R package Startrac (version 0.1.0) [19].

### Target expression in public datasets

Single-cell tumor-infiltrating immune cell atlas from 13 cancer types [21] was used to visualize target gene expression. To evaluate expression in our previously published GC dataset [18], we reference mapped T and NK cells to the tumor-infiltrating immune cell atlas as outlined above. For visualization in both datasets, all CD4 subtypes were grouped as Naïve cells, T Helper cells were renamed as “CD4_TFh,” cytotoxic and dysfunctional subsets were grouped as described above.

### Multiplex immunofluorescence

Antibodies used for multiplex immunofluorescence (mIF) staining included CD8α (C8/144B, #70306, 1:800), TIGIT (E5Y1W, #99567T, 1:800), FOXP3(D2W8E, # 98377, 1:200), GITR (D9I9D, #68014, 1:400), and Signal Stain Boost IHC Detection reagents for species-specific HRP conjugated secondary antibodies (all from Cell Signaling Technology). TSA Plus Fluorescein, Cyanine 5, and Cyanine 3 kit (Akoya Biosciences) were used for tyramide signal amplification. Staining was carried out as per the manufacturer’s protocol (Cell Signaling Technology). Briefly, FFPE sections were deparaffinized in Histochoice clearing agent and hydrated in a descending alcohol series. Antigen retrieval was performed with boiling 1 mM EDTA, pH 8.0 using a microwave with maintenance at a sub-boiling temperature for 15 min. Staining order, antibody concentrations, and fluorophore combinations were optimized using a human tonsil section obtained from the Stanford Tissue Bank. Order of antibodies and fluorophores in one panel was GITR (Cy3), FOXP3 (Cy5), and CD8 (Fluorescein) used sequentially. Another panel comprised TIGIT (Cy3), FOXP3 (Cy5), and CD8 (Fluorescein) used sequentially. Stripping of antibodies following signal amplification was performed using boiling 10 mM sodium citrate, pH 6.0 in a microwave followed by maintenance at sub-boiling temperature for 10 min and cooling on bench top for 30 min. Nuclear staining was performed with 2 μg/ml DAPI (Thermo Fisher Scientific).

### RNA in situ hybridization

RNA in situ hybridization (RNA-ISH) was performed for *GZMB* using the RNAscope Multiplex Fluorescent Reagent kit v2 (ACD BioTechne) as per the manufacturer’s protocol for FFPE sections. TSA Plus Cyanine 5 was used for detection. Staining pattern was confirmed in a human tonsil section. Positive and negative control probes supplied by the manufacturer were used to evaluate signal to noise ratio in the tonsil section.

### Image analysis

Images were acquired on a Zeiss Axio Imager Widefield Fluorescence Microscope (Stanford Neuroscience Microscopy Service) from two or three representative regions of interest per sample. Image analysis was performed in QuPath version 0.3.2 [36]. Cell detection was performed with default parameters except minimum area was set to 5 μm^2^. Composite classifier was created for mIF staining in each sample using mean signal intensity thresholds per cell for each fluorophore. Steps outlined in the QuPath vignette were followed. Cells positive for both FOXP3 and CD8 were filtered (1.88–2.67% of total cells). For RNA-ISH analysis, number of spots per cell was counted using subcellular detection function in QuPath.

### Additional statistical analysis and visualization

We used the Adjusted Rand Index (ARI) to compare similarity between cluster labels and condition batch meta data label for each cell. A vector of these respective class labels was supplied to the “adjustedRandIndex” function in mclust package (version 5.4.7). Additional analysis or visualization was conducted using R packages stats (version 4.1.0.), tibble (version 3.1.7), dplyr (version 0.7.6), broom (version 0.7.6), ggplot2 (version 3.3.6), ggpubr (version 0.4.0), and ComplexHeatmap (version 2.9.3) [37] in R version 4.1.0 [38]. Seurat functions “DimPlot,” “DotPlot,” and “FeaturePlot” were also used for visualization. Figures were additionally edited in Adobe Illustrator CS6 (version 16.0.0).

## Results

### Experimental approach and study design

We obtained ten surgical resections of CRCs or GCs from eight different patients (Table 1, Additional file 1: Table S1). They comprised four male and four female patients with an average age of 62 years. The tissue samples included seven resections of primary CRC from seven individual patients. From one patient with GC, we obtained three independent resections—one from the primary tumor and two from metastases to the peritoneum, which is an organ that lines the abdominal cavity.

**Table 1:** Study samples

		Experimental conditions				
Sample ID	Tumor site	T0	ctrl	PMAIono	GITR	TIGIT
CRC-1	Primary CRC	+	+	+	+	-
CRC-2	Primary CRC	+	+	+	+	-
CRC-3	Primary CRC	+	+	+	+	-
CRC-4	Primary CRC	+	+	+	-	+
CRC-5	Primary CRC	+	+	+	+	+
CRC-6	Primary CRC	+	+	+	+	-
CRC-7	Primary CRC	+	+	+	+	+
GC1-1	Primary GC	+	+	+	+	-
GC-1-2	Metastatic GC	+	+	-	+	+
GC-1-3	Metastatic GC	+	+	-	+	+

*CRC* colorectal cancer, *GC* gastric cancer, *T0* original surgical resection, *ctrl* control, *PMAIono* PMA Ionomycin

The samples underwent rapid processing following surgical resection (Methods). From each tumor, we split the tissue, using one portion to generate single-cell suspensions and scRNA-seq and scTCR libraries for sequencing (Fig. 1A, “Methods”). The tissues that were processed immediately into single-cell libraries provide a baseline of the cellular composition of the tumor. We refer to this baseline as time-point zero (“T0”). The other portion was used for TSC culturing. These results were used for determining cellular changes that may occur during the TSC culturing.

For experimental testing of each cancer’s TME, we generated ex vivo tumor slice cultures (TSCs) from the resections. There were four different conditions. The TSCs were treated with either (i) isotype control antibody (“ctrl”), (ii) known T cell activator PMA/Ionomycin (“PMAIono”) as a positive control, (iii) GITR agonist antibody (“GITR”), or (iv) TIGIT antagonist antibody (“TIGIT”). After 24 h of treatment, the cells were harvested and then processed for scRNA-seq and scTCR-seq. The number of experimental conditions tested per sample depended on the available size of each resection. All ten samples had adequate tissue for a baseline T0 and control sample for scRNA-seq. We conducted scRNA-seq on PMA/Ionomycin treatment from eight samples, GITR agonist treatment from nine samples, and TIGIT antagonist treatment from five samples (Table 1). Quality control measures including filtering cells for mitochondrial genes indicative of cell death [39] and doublet identification [24]. Following filtering, our final analysis included a total of 236,483 single cells with an average of 5630 cells per sample (Additional file 1: Table S2).

### Baseline immune cell characteristics of the TME from primary gastrointestinal tumors

We determined the baseline cellular composition of the T0 samples. Batch effects were reduced using the Harmony algorithm [25]. Specific cell type clusters were composed of different samples, indicating the elimination of batch effects (Fig. 1B). Using the scRNA-seq data, we made cell type assignments based on canonical marker genes (Methods). Overall, we identified tumor epithelium, myeloid cells, stromal cells, and lymphocytes. We sub-clustered each major lineage to characterize cellular features in greater detail. For these results, we denote the cell type and functional state by listing prominent examples among the associated gene expression markers.

Tumor epithelial cells expressed well-characterized markers of CRC or GC [18, 40] (*KRT7*, *KRT17*, *ELF3*, *CEACAM6*, *FABP1*, *FABP5*, *SPINK1*, *REG4*, *TFF3*) (Additional file 2: Fig. S1A, S1B). Stromal cells included fibroblast subsets with expression of extracellular matrix-related marker genes including *MGP*, *DCN*, and Collagen family genes (Fig. 1C, Additional file 2: Fig. S1C). We also detected smooth muscle cells (*ACTA2*, *TAGLN*) and pericytes (*RGS5*, *PDGFRB*, *NOTCH3*). Peritoneal metastasis samples (GC_1_2, GC_1_3) also contained mesothelial cells (*SLPI*, *UPK3B*, *KRT8*, *KRT18*, *KRT19*) [40, 41]. Endothelial cells expressed known arterial, venous, capillary (*PLVAP*, *VWF*, *CD320*, *PECAM1*, *KDR*, *ENG*, *ACKR1*, *SELE*, *ICAM2*, *SRP14*, *SRGN*), or lymphatic markers (*CCL21*, *LYVE1*, *PROX1*, *PDPN*) [18, 40, 42].

Myeloid lineage cells included macrophages, dendritic cells (DCs), and mast cells (Fig. 1D, Additional file 2: Fig. S1D). Among macrophages we detected previously characterized subsets [21, 43, 44] including infiltrating monocytes (*S100A8*, *S100A9*, *FCN1*, *VCAN*), proinflammatory (*CXCL8*, *IL1B*, *IL6*, *IL8*), anti-inflammatory LYVE1+ (*LYVE1*, *FOLR2*, *PLTP*), SPP1+ (*SPP1*, *APOE*, *TREM2*, *CTSB*, *MMP9*), and C1QC+ (*C1QA*, *C1QB*, *C1QC*, *APOE*) macrophages. DCs included conventional DCs (cDC) subsets (*CLEC9A*, *FLT3*, *IDO1*, *CD1C*, *FCER1A*, *HLA-DQA1*, *HLA-DQB1*, *LAMP3*, *CCR7*, *CCL22*, *CCL19*) as well as plasmacytoid DCs (pDC) (*GZMB*, *SOX4*, *JCHAIN*, *IRF7*) [18, 43, 45]. Mast cells highly expressed known marker genes (*TPSAB1*, *TPSB2*, *KIT*, *GATA2*, *CPA3*, *MS4A2*) [43].

Among lymphocytes, we detected B cells expressing B cell markers *MS4A1*, *CD79A*, *CD79B*, *CD19*, *CD83*, and *CD37* (Additional file 2: Fig. S1E, F) [21]. Plasma cells lacked mature B cell marker genes and expressed known marker genes including *SDC1*, *TNFRSF17*, and immunoglobulin genes including *JCHAIN*, *IGKC*, *IGHG1*, *IGHM*, and *IGLC2*. We also detected proliferating B cells expressing *MKI67*, *STMN1*, and *TUBA1B*.

We characterized the T and NK functional cell states using a method called cell reference mapping. This method used an established reference from a pan-cancer tumor immune cell atlas [21] and the SingleR algorithm [29]. Each cell’s gene expression is matched to a given reference cell type. This approach provides an unbiased identification of cell subtypes without applying cell clustering methods. We evaluated these cell states based on the expression of lineage markers, transcription factors, surface receptors, cytokine effectors, and other genes that have been extensively described in recent scRNA-seq studies [2, 20, 21].

We detected CD4 naïve cells (*CCR7*, *SELL*, *LEF1*, *TCF7, IL7R*) (Fig. 1E). We identified regulatory T (Treg) cells with high expression of *FOXP3*, *BATF*, *IL2RA*, co-stimulatory molecules *TNFRSF4* and *TNFRSF9*, and immune checkpoint *CTLA4*, resembling the profile of intratumoral Tregs identified by previous scRNA-seq studies [21]. Treg cells are immunosuppressive and limit anti-tumor activity through specific effects on cytotoxic CD8 T cells, dendritic cells, and macrophages [46]. As corroborated by other studies [35], we also observed a proliferative subset of Treg cells. These proliferative subsets may reflect a TME response to local tumor antigens [2].

We also detected *CXCL13* expressing CD4 T cells with low expression of T helper cell cytokines such as *IFNG*, *GZMA*, *GZMB*, *CCL3*, and *CCL5* [20] (Fig. 1E). These cells expressed several genes associated with T follicular helper (TFh) differentiation including transcription factors *NR3C1*, *TOX2*, *TOX*, *TSHZ2*, *RBPJ*, and *BHLHE40* but did not express *CXCR5* or *BCL6* [20]. They also expressed genes associated with CD8 exhaustion including *NMB*, *CD200*, and *PDCD1*. We also detected a proliferating subset of these cells with higher expression of T helper cytokines, possibly reflecting a response to tumor antigens [2]. These cells resemble previous scRNA-seq analysis findings which variously labelled them as CXCL13+ T helper-like cells [19], CD4_CXCL13 cells [47, 48], CD4- CXCL13 with TFh-like features [34], PD-1^+^CXCR5^−^CD4^+^Th-CXCL13 [49], dysfunctional TFh [35], TFh-related cells, or TFh/Th1 cells [20]. We refer to these cells as TFh-like cells. TFh-like cells have been linked to anti-tumor immunity by promoting CD8 and B cell activity [2, 50].

CD8 naïve cells (*CCR7*, *SELL*, *LEF1*, *TCF7*) lacked effector cytokine or checkpoint expression (Fig. 1F). We also detected NK cells with high expression of various cytotoxic effector genes including *NGK7*, *GNLY*, *PRF1*, *CCL4*, *GZMA*, *GMZB*, and *GZMH* together with Killer cell lectin-like receptors [21].

Among the CD8 T cells, we identified effector cytotoxic CD8 characterized by high expression of effector cytokine *GZMK* and low expression of immune checkpoints (Fig. 1F). *GZMK* expression in CD8 effector cells has been associated with early dysfunction [2, 20, 51]. We observed *CXCL13* expressing dysfunctional CD8 T cells with increased expression of inhibitory (*LAG3*, *PDCD1*, *HAVCR2*, *CTLA4*) and co-stimulatory (*TNFRSF9*) receptors [2]. These cells also expressed genes (*ENTPD1*, *LAYN*) and transcription factors (*RBPJ*, *TOX*, *PRDM1*) linked with exhaustion. Additionally, they continued to express cytotoxic effectors (including *GZMA*, *GZMB*, *GZMH*) reflective of their anti-tumor potential [20]. Dysfunctional CD8 T cells had a subset of proliferating cells (noted by expression of the marker genes *MKI67*, *STMN1*, *TUBA1B*) with intermediate *CXCL13* and checkpoint gene expression. Proliferating dysfunctional CD8 T cells have been linked to early dysfunction in a clonal tumor-reactive population [35]. Rare subpopulations of dysfunctional CD8 T cells function as precursor or progenitor cells that give rise to terminally exhausted cells [2]. We examined a set of genes linked to precursors of exhausted T (TPEX) cells (*TCF7*, *CCR7*, *SELL*, *IL7R*, *TNFRSF4*, *IL6R*, *IGFL2*) [43, 52] (Additional file 2: Fig. S1G). Rare cells in both dysfunctional and dysfunctional proliferating cells expressed TPEX genes. Hence, our analysis identified transitional states of CD8 T cell dysfunction in the TME including progenitor, early, and late dysfunction that have been previously reported [2].

These cell types were identified across all patients in varying proportions (Additional file 1: Table S3). In summary, across all tissue samples, the TME in the baseline T0 resections contained diverse functional T cell states with anti-tumor (cytotoxic CD8, dysfunctional CD8, TFh-like) and immunosuppressive (Treg) properties.

### Baseline T cell receptor clonality in the primary tumor TME

To assess clonality of the T cells in the cancer’s TME at the baseline state, we performed scTCR-seq on the baseline T0 samples. We identified TCR chains from an average of 57% of the T cells with matching single-cell gene expression (range 31–78%). Next, we determined whether there was evidence of TCR clonotypes being highly represented within a given sample [19]. This overrepresentation is termed as being “an expansion” for a given T cell clonotype. Moreover, one can assign specific clonotypes to different transcriptional cell states (i.e., Tregs, Tfh) using matched cell barcodes.

**Fig. 2:**
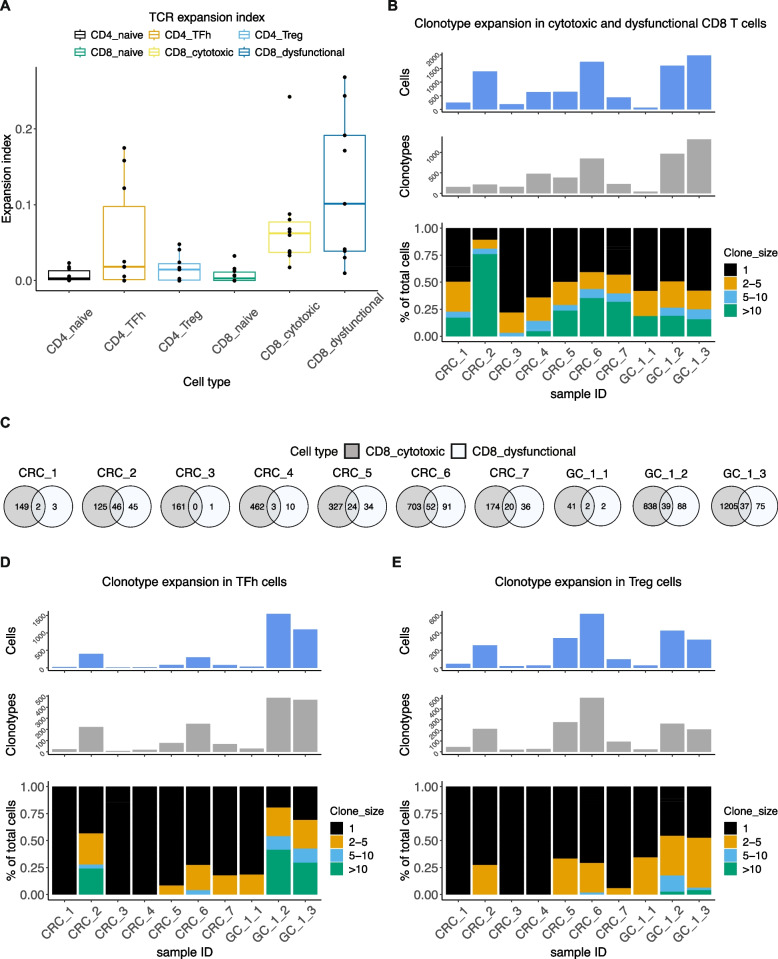
**A** TCR expansion index for respective cell types. **B** Frequencies of clonotypes in CD8 T cells from respective patients together with absolute number of cells and clonotypes examined. **C** Overlap between TCR clonotypes in cytotoxic and dysfunctional CD8 T cells from respective patients. **D**, **E** Frequencies of clonotypes in **D** TFh-like and **E** Treg cells from respective patients together with absolute number of cells and clonotypes examined

To conduct this analysis, the frequency of individual clonotypes was calculated using the Shannon entropy score—this metric quantifies T cell clonotype expansion with a value range of 0 to 1, with 1 indicating high clonality. Cytotoxic and dysfunctional CD8 T cells showed high expansion index of TCR clones (Fig. 2A). High clonality and expansion may be an indicator of tumor antigen-driven expansion in the TME [19]. Next, we examined the frequency distribution of CD8 T cell clonotypes across samples (Fig. 2B). Single-cell clonotypes represented the majority of TCRs, indicating a lack of expansion among these clonotypes. Across the samples, between 22 and 89% of the total cells were represented by expanded clonotypes.

We investigated the overlap between clonotypes found in cytotoxic and dysfunctional CD8 T cells (Fig. 2C). We detected an average overlap of 36% (range 23 to 52%) between the two cell types across all samples. This analysis excluded tumor CRC_3 with only one clonotype detected in dysfunctional CD8 T cells. Hence, a subset of *GZMK*+ effector cells were linked to the dysfunctional phenotype in agreement with previous studies [2, 20, 35].

CD4 TFh-like and Treg cells had a low degree of expansion in a subset of samples (Fig. 2A). Examining the clonotype frequency distribution confirmed that three out of ten tumors did not contain expanded clones (Fig. 2D, E). In the remaining seven tumors, expanded clonotypes comprised between 8 and 80% of all cells in TFh-like and 6–55% in Tregs. Although the total number of cells analyzed affected these expansion metrics, our findings resemble previous studies, which detected highest expansion in CD8 T compared to TFh-like and Treg cells [20, 35].

Overall, this analysis identified TCR sequences of expanded clones in infiltrating T cells that may be potentially tumor-reactive in each sample at baseline.

### GITR and TIGIT gene and protein expression in the baseline TME

**Fig. 3:**
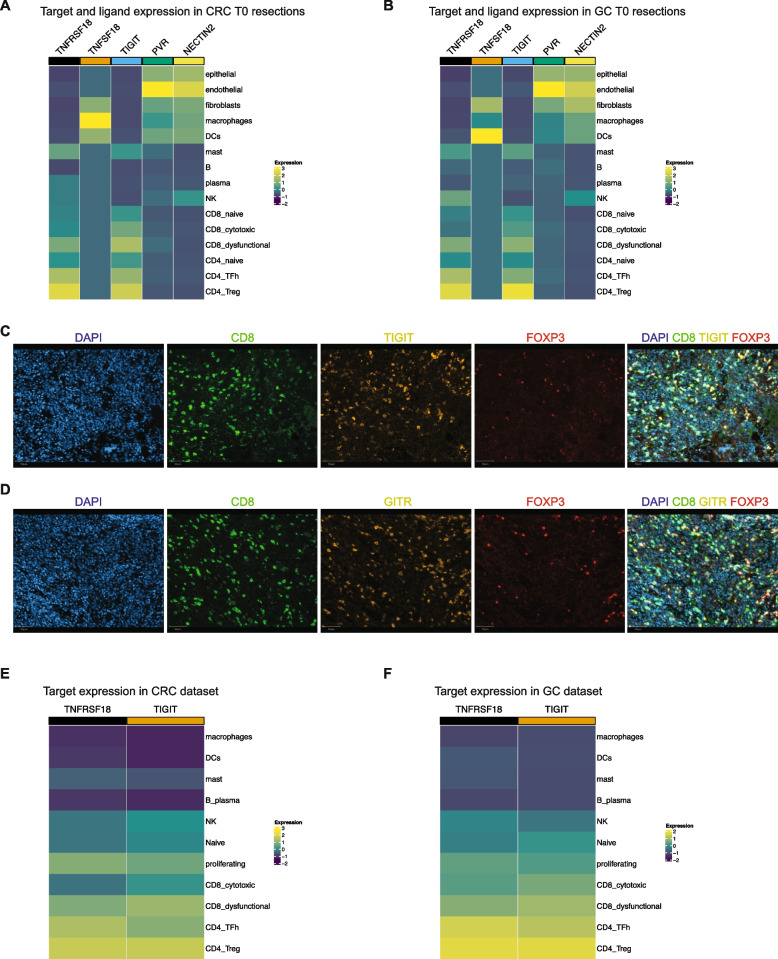
**A**, **B** Scaled average expression of respective genes in various cell types from **A** all CRC T0 resections and **B** all GC T0 resections. **C**, **D** Immunofluorescence staining for respective proteins or their merged image in an example region of interest from sample CRC-2. Scale bar = 50 μm. **E**, **F** Scaled expression of respective genes in various cell types from **E** CRCs in the publicly available tumor immune atlas dataset and **F** our previously published GC dataset

We evaluated the gene expression of the immunotherapy targets *TNFRSF18* (encoding protein GITR) and *TIGIT* among gastrointestinal cancers at the baseline state. In both CRCs and GC tumors, the dysfunctional CD8, TFh-like, and Treg cells had the highest levels *TNFRSF18* expression (Fig. 3A, B). The complementary ligand, *TNFSF18,* encoding the protein GITRL, was expressed by fibroblasts, DCs, and macrophages in CRC. *TIGIT* expression was highest in cytotoxic CD8, dysfunctional CD8, TFh-like, and Treg cells. The genes *PVR* and *NECTIN2*, which encode for TIGIT ligands, were expressed by tumor epithelial, endothelial, fibroblasts, macrophages, and DCs in the TME. These expression patterns are along the lines of other reports [53, 54]. Overall, this result indicated that among all samples, the TME cells expressed genes required for GITR and TIGIT receptor-ligand signaling.

We also measured the protein expression of GITR and TIGIT in these baseline tumor tissues (T0 resections) using multiplexed immunofluorescence (mIF) staining. We used two independent antibody panels containing CD8, FOXP3, and TIGIT or CD8, FOXP3, and GITR respectively (Fig. 3C, D). We performed image analysis using a multiplex classifier for detecting single-stain or double-stain positive cells as described previously [18]. From all samples, an average of 37.4% of total CD8 positive cells expressed TIGIT. An average of 53.68% of total FOXP3 positive cells were TIGIT positive (Additional file 2: Fig. S2A, B). Similarly, 42.46% of CD8 cells expressed GITR (Additional file 2: Fig. S2C, D). Among the FOXP3 cells, 74.5% expressed GITR. These results confirmed that among our tumor samples, CD8 T cells and Tregs expressed the TIGIT and GITR protein.

Overall, these results indicated the TME expression of the target receptors and their ligands among the tumors that were used for these experiments. Targeting these receptors has the potential to modify the function of anti-tumor T cell subsets such as cytotoxic CD8, dysfunctional CD8, and TFh-like cells, as well as immunosuppressive Tregs.

### Expression of TIGIT and GITR in colorectal and other cancer types

To determine the expression of these two targets among an expanded, independent set of colorectal, gastric, and other tumor types, we analyzed gene expression among a data set of 13 different cancer types [21]. Importantly, this dataset included 25 independent CRCs (Fig. 3E). For gastric cancer, we evaluated our previously published dataset of seven GC samples [18] (Fig. 3F). High *TNFRSF18* and *TIGIT* expression was detected in dysfunctional and cytotoxic CD8 T, TFh-like, Treg, and proliferating cells confirming results from CRC and GC T0 samples.

Other cancer types included breast carcinoma (BC), basal cell (BCC) and squamous cell carcinoma (SCC), endometrial adenocarcinoma (EA), renal cell carcinoma (RCC), intrahepatic cholangiocarcinoma (ICC), hepatocellular carcinoma (HCC), pancreatic ductal adenocarcinoma (PDAC), ovarian cancer (OC), non-small-cell lung cancer (NSCLC), and cutaneous (CM) and uveal melanoma (UM) (Additional File 2: Fig. S2E, F). Across all cancer types, target expression followed similar patterns as GC and CRC. In EA and OC, high *TNFRSF18* expression was noted in DCs and NK cells respectively. These results confirmed the expression of these targets among CRC and GC tumors as well as a wide variety of solid tumor types.

### Primary tissue slice cultures maintain the native TME composition of gastrointestinal cancers

As noted previously, tissue slice cultures have been demonstrated to maintain a high degree of tissue viability, cellular diversity, and cellular transcriptional profiles [6, 10]. We integrated data from all ex vivo ctrl and treatment experiments and performed cell type identification using marker based and SingleR assignments (Methods).

First, we confirmed that the TSCs cultured for 24 h maintained the cellular characteristics similar to the baseline state of the TME (i.e., T0 cell conditions at time of resection). As noted previously, other groups have used short culture periods to maintain the cellular diversity found in the native TME [4–7]. We evaluated the TSC cellularity using hematoxylin and eosin (H&E) staining of the cultures. This result showed that cell morphology remained intact with little evidence of necrosis or other signs of overt cell death (Additional file 2: Fig. S2G).

**Fig. 4:**
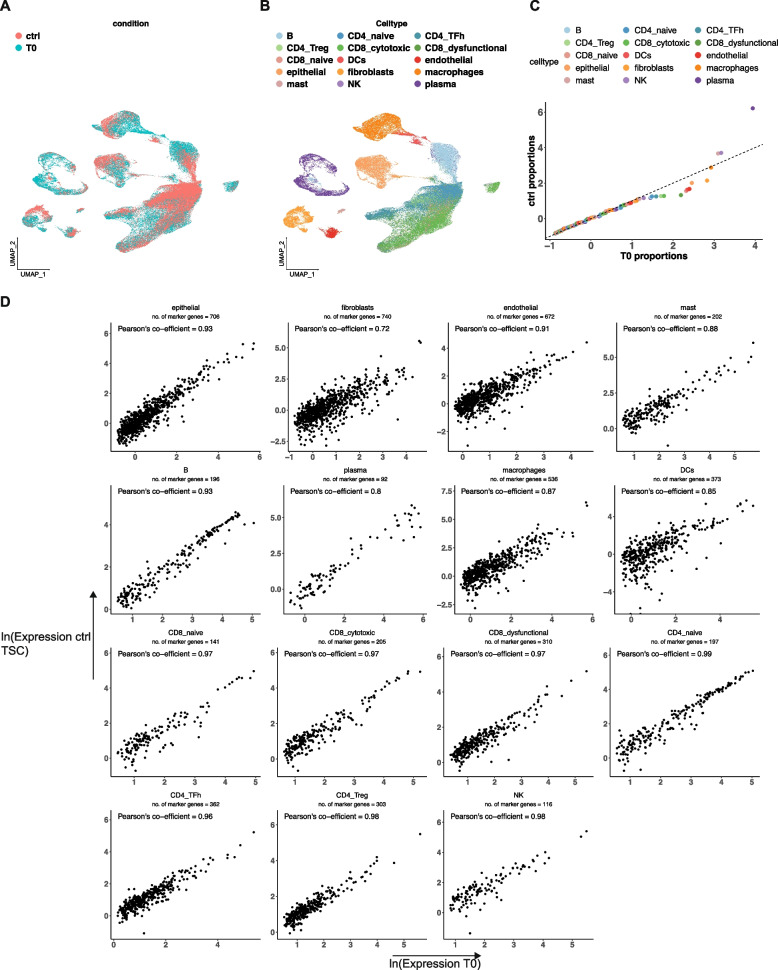
**A**, **B** UMAP representation of dimensionally reduced data from T0 and 24 h ctrl TSCs following batch-corrected graph-based clustering of all datasets colored by **A** experimental condition and **B** cell type. **C** Quantile-quantile plot comparing the proportion distributions of respective cell lineages across all T0 and ctrl TSCs. **D** Scatter plot indicating average log expression of marker genes for T0 cell lineages in T0 and ctrl TSC in respective cell lineage, annotated with the number of marker genes examined. Pearson’s co-efficient was calculated using non-log transformed values

Next, we evaluated the single-cell gene expression across the two conditions for all samples which included: (i) the baseline T0; (ii) TSC following a 24 h incubation with isotype control antibody; (Fig. 4A). We corrected the data for experimental batch but not for the experimental condition, using the Harmony algorithm [25]. Cells belonging to the baseline T0 tissue and control clustered together in the Uniform manifold approximation and projection (UMAP). This result indicated that the cells had similar gene expression profiles. We calculated the Adjusted Rand Index (ARI) to determine the variation in gene expression between the baseline and control culture. Cluster labels compared to the experimental condition had a low ARI value of 0.009 which indicated that clustering was due to the cells having similar gene expression characteristics and not driven by the T0 or TSC experimental condition. Despite the low ARI, we observed shifts in the UMAP embeddings between the two conditions. This indicated that transcriptional profiles in TSCs resemble T0 but are not completely identical.

We compared cell types in T0 and TSC samples. The TSC samples contained all cell types compared to the matched baseline T0 samples. These cell types included tumor epithelial, macrophages and dendritic cells, NK, B or plasma lymphocytes, mast cells, fibroblasts, and endothelial cells, as well as T cell subsets including naïve, cytotoxic CD8, dysfunctional CD8, Treg, and TFh-like cells (Fig. 4B). The relative proportion of all cell lineages was also maintained in the TSCs compared to the baseline tumor tissue (Fig. 4C).

We identified differentially expressed genes for each cell lineage in the baseline T0 samples (Seurat Wilcoxon test, log_2_ fold change ≥ 0.4, adjusted *p* ≤ 0.05). We compared the average gene expression of these genes in each respective cell lineage in T0 to TSCs. Expression was highly correlated across all cell types (Fig. 4D) (Pearson correlation ≥0.72, *p* ≤ 7.1E-22). Hence, the TSCs maintained the cellular heterogeneity and closely resembled the transcriptional cell states that were present in the original tumor.

### General stimulation of T cells and other cell types in the TSC TME

To demonstrate that the TSC cells were functionally responsive, we used an activation control stimulus with phorbol ester 12-myrisate 13-acetate and the calcium ionophore ionomycin (PMA/Ionomycin). In combination, these compounds stimulate downstream pathways associated with T cell activation and have been extensively studied [55]. We evaluated specific subsets of cells from samples treated with ctrl and each respective perturbation. To account for interpatient variability in differential expression (DE) analysis, we utilized model-based analysis of single-cell transcriptomics (MAST) [30] incorporating sample as a random effect in the model [31]. A threshold of log_2_ fold change of 0.4 and false discovery rate (FDR) *p* < 0.05 was used to identify significantly DE genes.

**Fig. 5:**
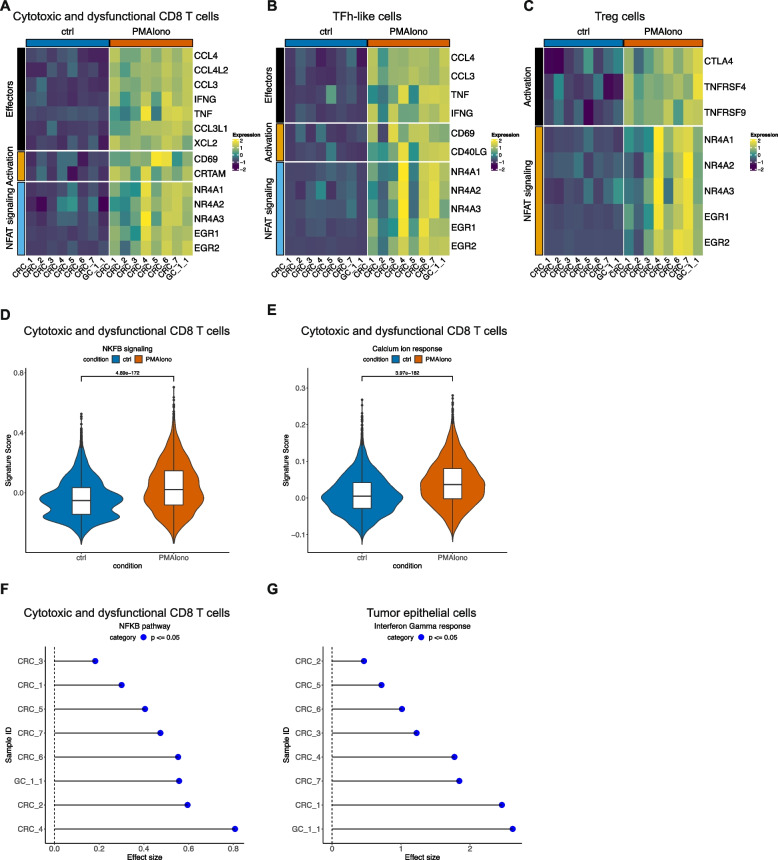
**A**–**C** Scaled average expression of respective genes in control or PMA/Ionomycin-treated samples in **A** CD8 T cells, **B** TFh-like cells, and **C** Treg cells. **D**, **E** Respective pathway activity in control and treated CD8 T cells with *T*-test *p*. **F**, **G** Cohen’s effect size and *p* of *t*-test comparison of respective pathway activity between control and treated cells from each individual sample

From the TSCs exposed to PMA/Ionomycin, we detected differentially expressed genes associated with activation in CD8 T cells (*CD69*, *CRTAM*) (Fig. 5A), TFh-like cells (Fig. 5B) (*CD69*, *CD40LG*), and Tregs (*CTLA4*, *TNFRSF4*, *TNFRSF9*) (Fig. 5C). All cell types demonstrated a response with increased expression of NR4A and EGR family genes which are associated with signaling of the nuclear factor of activated T cells (NFAT). This pathway is associated with T cell activation and anergy following stimulation [56]. In CD8 T and TFh-like cells, we also identified increased expression of several effector cytokines and chemokines including *CCL4*, *CCL3*, *IFNG*, and *TNF* relative to control. These effects were observed across all tumors. At the pathway level, we confirmed a significant increase in NF-KB signaling and calcium ion response pathway activity in CD8 T cells (Fig. 5D, E). Both pathways are known mediators of effects of PMA/Ionomycin [55]. Across all the tumors, CD8 T cells consistently responded to PMA/Ionomycin stimulation as indicated by a significant increase in NF-KB activity (Fig. 5F).

The tumor epithelial cells showed significant increases in interferon (IFN) gamma response—signaling across all tumor samples (Fig. 5G). This indicated that increased IFN from activated T cells was able to affect neighboring tumor cells, reflecting the preserved intercellular networking in TSCs. Overall, these experiments with PMA/Ionomycin confirmed that the TSC cells were functional and demonstrated intercellular interworking.

### GITR activation had limited and heterogenous effects on CD8 T cell cytotoxicity

**Fig. 6:**
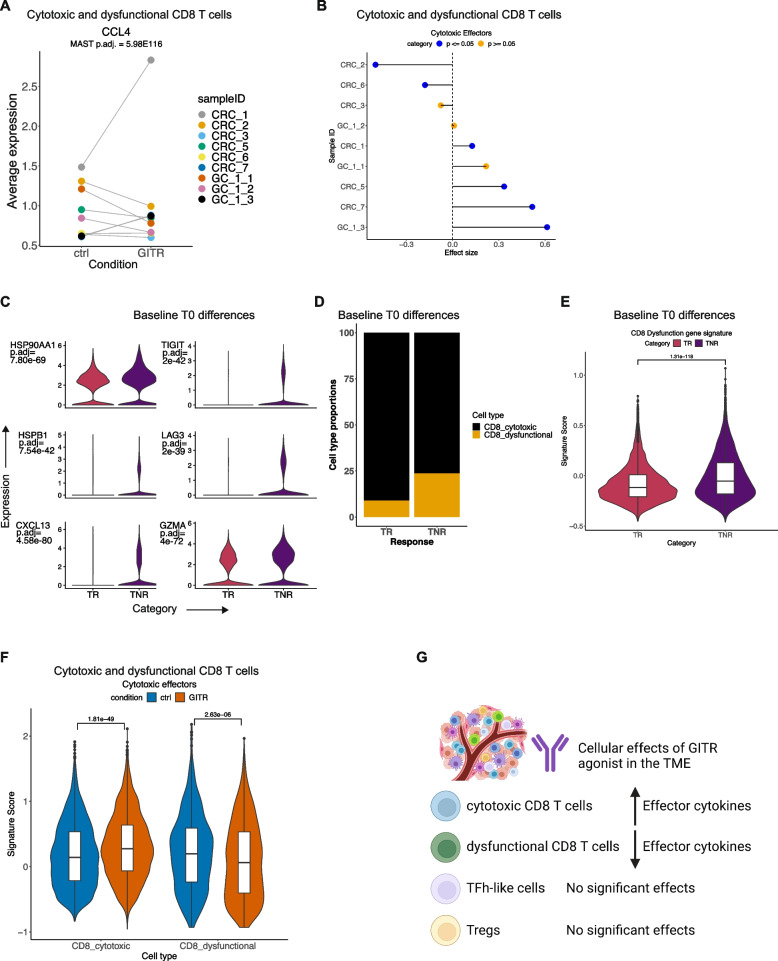
**A** Average expression of *CCL4* in each sample in control and treated CD8 T cells with MAST DE adjusted *p*. **B** Cohen’s effect size and *p* of *t*-test comparison of cytotoxic effector pathway activity between control and treated CD8 T cells from each individual sample. **C** Expression of respective genes in cells from TR or TNR baseline T0 samples with Seurat Wilcoxon adjusted *p*. **D** Proportions of CD8 T cell subtypes in baselineT0 samples corresponding to transcriptional responders (TR) or non-responders (TNR). **E** Expression of gene signature of CD8 T cell dysfunction in TR and TNR with t-test *p*. **F** Cytotoxic effector pathway activity in control and treated cytotoxic and dysfunctional CD8 T cells with *t*-test *p*. **G** Schematic representation summarizing the ex vivo effects of GITR agonist in the TME

We evaluated the effects of the GITR agonist on the TSCs. For cytotoxic and dysfunctional CD8 T cells, the only gene which showed significant differential expression was *CCL4*. This gene had a fold change of >0.4 upon GITR agonist treatment (MAST DE with sample as random effect) (Fig. 6A). Strongest increase in *CCL4* expression was observed in CRC-1, with slight increases in CRC-6, CRC-7, and GC-1-3. Other significantly increased genes with lower fold changes (>0.15, FDR < 0.05) included cytokines *CCL4L2*, *GZMA*, *GNLY*, *CCL3*, and *PRF1* (Additional file 1: Table S4). Thus, GITR agonist exposure had limited effects on gene expression across the tumors, with interpatient variability.

We evaluated the effect of GITR agonist on the expression of a CD8 T cell cytotoxic gene expression signature that has been previously reported [33, 34] (Additional file 1: Table S5, Fig. 6B). Significant increases in cytotoxic gene signature expression were observed in four (CRC-1, GC-1-3, CRC-5, CRC-7) out of nine tumors. Hence, the GITR agonist showed interpatient variability in terms of a gene expression response.

### Dysfunctional CD8 T cells were indicators of no response to the GITR agonist

We further investigated these differences in transcriptional responsiveness to GITR agonist. GITR agonist exposure led to only a limited increase in cytotoxic effectors for only a subset of the tumors. We grouped tumors as transcriptional responsive (TR) when they responded with an increase in cytotoxic effector gene expression upon treatment (GC-1-3, CRC-1, CRC-5, CRC-7). Tumors lacking this response were identified as transcriptional non-responsive (TNR) (GC1-1, GC-1-2, CRC-2, CRC-3, CRC-6).

We compared the baseline CD8 T cells (T0) in the TNR versus TR samples. No differences were observed in the TCR clonotype characteristics of these samples (Fig. 2B). We identified differentially expressed genes between the TNR and TR samples. The TNR-associated cells had significantly increased expression (Seurat Wilcoxon adjusted *p* < 0.05) of CD8 T cell dysfunction markers including *CXCL13*, *TIGIT*, and *LAG3* [20] (Fig. 6C). Additionally, these cells had increased expression of effector *GZMA* and HSP family genes that have been linked to exhaustion [57]. TNR samples also had a significantly higher proportion (23.7%) of CD8 dysfunctional cells than TR (8.9%) (Fig. 6D) (two proportions *z*-test *p* < 2.2e−16). Cytotoxic CD8 cells were increased in TR relative to TNR. We also evaluated a gene signature associated with CD8 T cell dysfunction that has previously been described [35] (Additional file 1: Table S5). TNR-associated cells had significantly higher levels of dysfunction (Fig. 6E). These results indicated that CD8 T cells with this dysfunctional phenotype do not respond to GITR agonist.

To test this association between a lack of transcriptional response in dysfunctional CD8 T cells, we examined ex vivo responses among CD8 T cell subsets across all tumors (Fig. 6F). Increased effector gene signature upon treatment was restricted to cytotoxic CD8 T cells. In dysfunctional cells, GITR agonist reduced effector gene expression. Hence, GITR agonist only stimulated effector cytotoxic cells. However, in exhausted dysfunctional cells, this stimulation reduced the cytotoxic potential.

Among TFh-like cells, the GITR agonist led to a significant increase in gene expression of only *S100A4*. No significant changes were detected in Tregs and NK cells (Additional file 1: Table S6-S8). These results indicated a limited effect of GITR agonist in the TME (Fig. 6G).

### TIGIT inhibition activated CD8 T cells in the TME

**Fig. 7:**
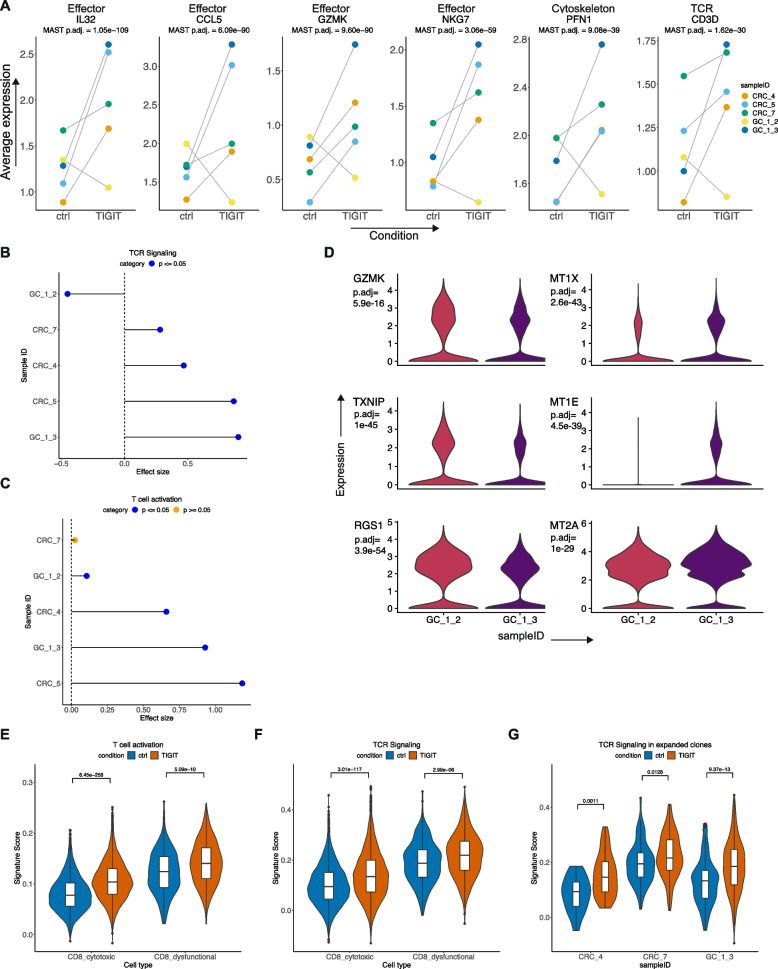
**A** Average expression of respective genes in each sample in control or TIGIT inhibitor-treated CD8 T cells with MAST DE adjusted *p*. **B**, **C** Cohen’s effect size and *p* of *t*-test comparison of respective pathway activity between control and treated cells from each individual sample. **D** Violin plots depicting the expression of respective genes in CD8 T cells from GC-1-2 and GC-1-3 samples with Seurat Wilcoxon adjusted *p*. **E**–**G** Respective pathway activity in control and treated CD8 T cells with *t*-test *p* in **E**, **F** CD8 T cell subtypes and **G** baseline expanded CD8 TCR clonotypes per sample

Next, we evaluated the effects of the TIGIT antagonist on the TSCs of five tumors (CRC-4, CRC-5, CRC-7, GC-1-2, GC-1-3). Based on the target expression patterns we identified (Fig. 3), we began by analyzing effects on cytotoxic and dysfunctional CD8 T, TFh-like, and Treg cells, which had highest TIGIT expression. In addition, we assessed DCs, which can be modulated by TIGIT binding [58]. CD8 T cells showed increased expression of several cytotoxic effector genes (Fig. 7A, Additional file 1: Table S9). These genes included *IL32*, the granzyme family genes, *NKG7*, and *CCL5*. We identified increased expression of genes involved in actin cytoskeleton remodeling including *PFN1*, *COTL1* and *CORO1A*. The *CD3D* gene, a component of the TCR, increased upon TIGIT inhibition. Notably, these increases were observed for all tumors except GC_1_2. Overall, TIGIT inhibition increased TCR signaling and activation of CD8 T cells.

### TIGIT inhibition generated a variable cellular response in a metastatic gastric cancer

Using gene signatures of TCR signaling or T cell activation, we examined tumor-specific responses to TIGIT inhibition. The CD8 T cells from all tumors responded with a significant increase in either one or both processes of TCR signaling and T cell activation upon treatment (Fig. 7B, C). We validated the significant increase in expression of downstream cytotoxic effector *GZMB* using RNA in situ hybridization (RNA-ISH) in tumors CRC-5 and GC-1-3 (Additional file 2: Fig. S3A, B). Hence, TIGIT inhibition activated local infiltrating CD8 T cells in the TME across all tumors.

A notable response pattern was observed for tumors GC-1-2 and GC-1-3 (Fig. 7A–C). The GC-1 tumor samples represented patient matched pairs of peritoneal metastases (Table 1). Interestingly, there was a variation in the response among these two metastases. Compared to GC-1-3, tumor GC-1-2 had a lower increase in the extent of T cell activation and responded with a decrease in TCR signaling upon treatment. This indicated a reduced responsiveness to TIGIT inhibition in CD8 T cells in GC-1-2 compared to GC-1-3.

To determine the factors leading to variation in transcriptional response from two metastatic tumors, we examined differences in their baseline T0 CD8 phenotypes (Seurat Wilcoxon adjusted *p* < 0.05). GC-1-2 and GC-1-3 did not differ in their baseline TCR clonotype characteristics (Fig. 2B). GC-1-2 CD8 T cells had significantly higher expression of *GZMK* (Fig. 7D) associated with effector memory CD8 cells [2] and *RGS1* associated with pre-exhausted and exhausted CD8 T cells [59]. The reduced responsive cells also had upregulated *TXNIP*, which has been demonstrated to reduce effector functions in CD8 T cells in viral infection [60]. Conversely, GC-1-3 had increased expression of metallothionein genes *MT1E*, *MT1X*, and *MT2A*. In a recent study, metallothionein family genes were demonstrated to link levels of CD8 activation and dysfunction to modulate their effector capacity [61]. In summary, we identified that TIGIT inhibition had different effects across two metastatic gastric cancers from the same patient. Variation in TIGIT response was associated with genes that modulate effector, activation, and dysfunctional phenotypes among CD8 T cells.

### TIGIT inhibition activated dysfunctional CD8 T cells

We evaluated the effect of TIGIT antagonist among the different CD8 cell subtypes (i.e., cell states) by quantifying TCR signaling and T cell activation pathways. We observed significantly increased TCR signaling and T cell activation in both cytotoxic and dysfunctional CD8 T cells (Fig. 7E, F). This result indicated that TIGIT inhibition is capable of reinvigorating dysfunctional exhausted cells. In contrast, the GITR agonist reduced the cytotoxicity of dysfunctional cells.

### TIGIT inhibition activated specific CD8 clonotypes

TIGIT inhibition had specific effects on certain CD8 TCR clonotypes. From the baseline tumor tissue (T0), we identified TCR clonotypes in CD8 T cells across patients as previously described. Clonotypes that were present in more than one cell were indicative of potential tumor reactivity (Fig. 2B) [2]. We used these TCR clonotypes from the baseline to identify how the CD8 T cells with the same clonotype responded to TIGIT antibody versus the control in the TSCs. In three tumors, we recovered 10.6 -28.7% of these clonotypes in both the ctrl and TIGIT conditions, allowing us to examine the effect of treatment in these cells. TIGIT inhibition successfully increased TCR signaling among these clones (Fig. 7G). Among non-expanded clones, TIGIT inhibition had either no significant effect (CRC_4, CRC_7) or a smaller increase in TCR signaling (GC_1_3) (Additional File 2: Fig. S3C). This result indicated that TIGIT treatment can specifically increase the activation of potential anti-tumor clonotypes.

Next, we examined if TIGIT inhibition could lead to an expansion of these CD8 T cell clonotypes. Since these results could be influenced by sampling effects, we compared the STARTRAC expansion index in TIGIT-treated samples with both respective ctrl and T0 samples. With only 24 h of treatment, TIGIT inhibition had a trend towards increased expansion following treatment (ANOVA with Tukey Honest significant difference *p* ≤ 0.5) (Additional File 2: Fig. S3D).

### Comparison of perturbation effects in CD8 T cells

We compared the gene signatures induced by PMA/Ionomycin, GITR agonist, and TIGIT antagonist treatment in CD8 T cells. For this analysis, we examined responses in tumors CRC_5 and CRC_7, which were treated with all three perturbations (Table 1). In both cytotoxic and dysfunctional CD8 T cells, PMA/Ionomycin led to significantly increased expression of several effector cytokines and chemokines including *CCL4*, *CCL3*, *IFNG*, and *TNF*, and NR4A and EGR family genes (*NR4A1*, *NR4A3*, *EGR2*) (Seurat Wilcoxon adjusted *p* < 2.2e−16) (Additional File 2: Fig. S4A, 4B). This increase was not observed with GITR and TIGIT. In cytotoxic CD8 T cells, GITR agonist led to an increase in effectors including *CCL5*, *GNLY*, *NKG7*, and *IL32*, to a higher extent than TIGIT treatment. Conversely, TIGIT inhibitor led to an increase in several T cell activation and TCR signaling-related genes including *CD8A*, *CD8B*, HLA genes, *LCK*, and effectors *GZMK*, *PRF1*. These effects were not observed with GITR agonist. In dysfunctional CD8 T cells, only two genes *CCL5* and *TPI1* were increased with GITR agonist. Genes increased by TIGIT antagonist overlapped with the response in cytotoxic CD8 T cells. Overall, these results resemble our findings examining each individual perturbation. Namely, PMA/Ionomycin led to a consistent increase in NFAT signaling and effector gene expression in both cytotoxic and dysfunctional CD8 T cells. GITR agonist increased effector cytokines in cytotoxic but not dysfunctional cells. TIGIT antagonist activated both cytotoxic and dysfunctional CD8 T cells with increased TCR signaling.

### TIGIT inhibition activated TFh-like cells in the TME

**Fig. 8:**
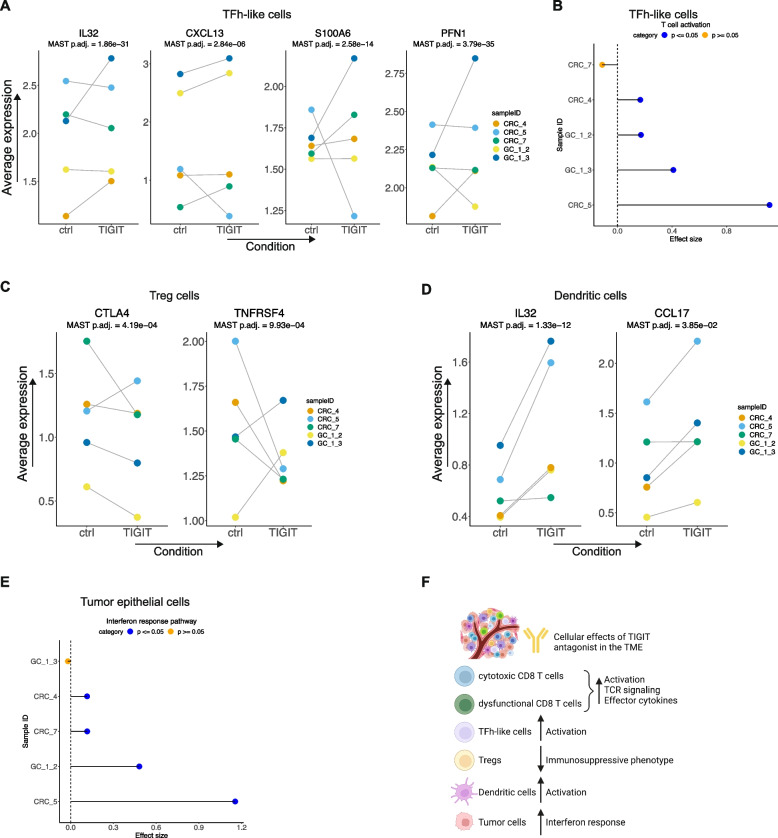
**A** Average expression of respective genes in each sample in control or TIGIT inhibitor-treated TFh-like cells with MAST DE adjusted *p*. **B** Cohen’s effect size and *p* of *t*-test comparison of pathway activity between control and treated TFh-like cells from each individual sample. **C**, **D** Average expression of respective genes in each sample in control or TIGIT inhibitor-treated **C** Treg cells and **D** DCs with MAST DE adjusted *p*. **E** Cohen’s effect size and *p* of *t*-test comparison of pathway activity between control and treated tumor epithelial cells from each individual sample. **F** Schematic representation summarizing the ex vivo effects of TIGIT antagonist in the TME

TIGIT inhibition led to activation of TFh-like cells, a cellular effect that has not been described previously. Differential expression analysis identified the upregulation of genes involved in T cell activation including *ACTB*, *PFN1*, *S100A4*, *S100A6*, and *TAGLN2* [62] (Additional file 1: Table S10, Fig. 8A). These cells also upregulated *IL32* expression, a cytokine with potential proinflammatory effects. Expression of *IL32* in the TME has been associated with response to PD-1 inhibition [63]. Importantly, TIGIT inhibition led to the increased expression of *CXCL13*. TFh-like cells which express CXCL13 may be associated with B cell response and generation of tertiary lymphoid structures [64]. These features mediate an effective immune response against a tumor. These effects were confirmed at the pathway level where TIGIT antagonist treatment led to a significant increase in the T cell activation ontology program. This effect was observed in four (CRC-4, CRC-5, GC-1-2, GC-1-3) out of five patients (Fig. 8B).

We examined the effect of TIGIT inhibition in expanded clonotypes, which were detected in our T0 analysis (Fig. 2D). In GC_1_3, we recovered 47.6% of these clonotypes in both the ctrl and TIGIT conditions. TIGIT inhibition successfully increased TCR signaling among these clones. This effect was not observed in non-expanded clones (Additional File 2: Fig. S4C, D).

Increased TFh-like cells have been demonstrated to predict response to and are proposed to be a target of PD-1 immunotherapy [14, 65]. However, the effects of TIGIT targeting on these cells in the human TME have remained unknown. We demonstrated that TIGIT antagonist activated these cells in the local TME. This represented an important cellular mediator of response to TIGIT inhibition that can generate an inflammatory anti-tumor TME.

### TIGIT inhibition’s effects on other cell types in the TME

The TIGIT antibody had some notable effects among the Treg cells (Additional file 1: Table S11). We observed an increase in *CD7* linked to a mature Treg phenotype [66] and a significant trend towards increased *CST7* (log_2_FC 0.37, confidence interval: 0.59 to 0.16) associated with TCR signaling [67]. However, this was accompanied by a significant trend in reduction of *CTLA4* (log_2_FC −0.36, confidence interval −0.17 to −0.56) and *TNFRSF4* expression. A reduction in expression in either of these two genes was seen in all patients (Fig. 8C). Both these molecules are key regulators of an immunosuppressive Treg phenotype [68]. This indicated modest effects of TIGIT inhibition on Tregs with a reduction in immunosuppressive phenotype.

Impact of TIGIT inhibition on DCs included a significant increase in expression of *CCL17* and *MARCKSL1*—these genes are indicators of DC activation and a maturation phenotype [69]. Accompanying this activated DC phenotype was a significant increase in *IL32* expression (Additional file 1: Table S12, Fig. 8D). DCs which express IL32 can activate T cell responses in the TME [70]. Conversely *IL2RA*, which is associated with an immunosuppressive DC phenotype [71], was reduced with TIGIT inhibition. TIGIT antagonist thus led to activation of DCs in the TME. This result has potential implications in improving antigen presentation and T cell priming to orchestrate an anti-tumor response in the TME.

Exposure to the TIGIT antagonist did not lead to any gene expression upregulation among NK cells (Additional File 1: Table S13). Finally, we examined the effects in tumor epithelial cells. TIGIT treatment led to an increase in IFN response signature among four (CRC-4, CRC-5, CRC-7, GC-1-2) out of five tumors (Fig. 8E). Overall, these results indicated that the proinflammatory effects modulated by TIGIT in various cell types in the TME translated into initial favorable transcriptional responses at 24 h in tumor epithelial cells. This included activation of both cytotoxic and dysfunctional CD8 T, TFh-like cells, and DCs together with a reduced immunosuppressive phenotype in Tregs, which can promote a favorable inflammatory TME (Fig. 8F).

## Discussion

Many immunotherapy agents and combinations are being studied in clinical trials, often with disappointing results [72]. It is important to determine the cellular basis for how these agents work in a dynamic and complex TME. An analysis of the TME and its response to these agents is critical to prioritize targets, identify mechanisms of resistance, and design rational treatment combinations. Our experimental design allowed the interrogation of cellular and transcriptional mechanism of action of perturbations in the TME. This identified heterogenous cellular and patient responses to GITR and TIGIT immunotherapy in the TME of GC and CRC.

Despite promising results from preclinical models of T cell culture, mouse, and primate models, GITR agonists have shown no meaningful clinical responses in recent clinical trials [73–77]. Our results demonstrated that GITR agonist has limited activity in the TME, restricted to cytotoxic CD8 T cells that lack exhaustion features. Moreover, dysfunctional cells had a decrease in cytotoxic activity upon GITR stimulation. Given that PD-1 inhibitors act to re-invigorate exhausted CD8 T cells, our finding raises the possibility that combining them with GITR agonists might antagonize this effect. We also saw no effects on Treg reprogramming in the TME with GITR agonist. In clinical trials, GITR agonist-mediated depletion of Tregs in the peripheral blood or TME has been observed only in some patients [77]. Discrepancy in effects of GITR agonist in reductionist preclinical models compared to complex ex vivo models and clinical trials could be explained by additional inhibitory mechanisms operating in infiltrating GITR expressing cells in the TME. Additionally, previous studies have indicated that suboptimal receptor clustering could explain the limited effects of agonist antibodies directed against several TNFRSF family members including GITR [78–80].

Compared to GITR agonist, we saw widespread effects in different cell types in the TME with TIGIT inhibition. This included activation of CD8 T cells and TFh-like cells. Both these components can mediate anti-tumor immunity. Our observation that TIGIT inhibition can increase TCR signaling in expanded CD8 clonotypes suggests that tumor antigen-specific T cells could potentially be reinvigorated with treatment. Early reports have demonstrated some clinical responses with TIGIT monotherapy or in combination with PD-1 [81]. However, these responses are likely to be restricted to only a subset of patients [82]. We did observe variation in the extent of transcriptional responses in CD8 T cells in our samples, which was associated with differential baseline expression of *GZMK*, *RSG1*, *TXNIP*, and metallothionein family genes. An expanded study with greater number of samples and mechanistic studies will allow us to examine these correlates of response to TIGIT inhibition.

The development of biomarkers that can predict clinical response can be guided by these molecular correlates. Based on our results, responsive tumors should contain infiltrating CD8, TFh-like, and Treg cells expressing TIGIT, along with ligand expression in the TME. When assessing CD8 T cells, it is important to consider not only their overall abundance but also specific characteristics such as GZMK expression. These cells would also need to be spatially located in close proximity to the tumor. The baseline and on-treatment TCR clonotype characteristics can reflect the anti-tumor potential of activated CD8 T cells and should be evaluated as biomarkers. Our study revealed that inhibiting TIGIT resulted in an increase in *IL32* expression in CD8 T cells, TFh-like cells, and DCs, which could potentially serve as an on-treatment response biomarker. Interestingly, a previous study has linked IL32 expression in the TME with response to PD-1 inhibition [63]. Ex vivo testing will also be useful in evaluating mechanisms of resistance to treatment and evaluating rational combinations. For example, upregulation of additional immune checkpoints such as PD-1 could impede effective T cell activation. Macrophages and fibroblasts in the TME can also mediate resistance via various immunosuppressive mechanisms [83]. Future studies testing TIGIT combination therapies with PD-1, macrophage and fibroblast targets will further improve clinical translation.

TSCs remain viable for 1–2 weeks in culture [5]. We evaluated the short-term perturbation effects after exposure to specific antibodies. This feature enables culture in media free from cytokines such as IL-2 that are routinely used in maintaining T cells in culture for longer duration. As we have demonstrated previously, IL-2 can reprogram transcriptional T cell states [84]. Our approach allows the evaluation of cell states in the original TME. Most studies of immunotherapy agents lack this feature.

A limitation of TSCs is that they allow interrogation only of the local TME, but not of peripheral and lymph node immune responses. These elements are also important players in the clinical response to immunotherapy [85]. Although TSCs retained the gene expression profiles of T0 resections, they were not identical. This could result from spatial heterogeneity in tumors, sampling effect during TSC generation, culture conditions, or the effect of isotype control. Short-term readouts also do not capture remodeling of the TME that could occur over longer duration, including expansion of TCR clonotypes. However, a recent study demonstrated that short-term fragment culture responses to PD-1 inhibition were correlated with long-term clinical responses [3]. While it only assesses the local TME response, our experimental strategy fills an important gap in preclinical studies.

## Conclusions

Our study identified the cellular and transcriptional mechanisms of action of GITR and TIGIT immunotherapy in the TME of patients. We used an experimental design combining a preclinical model that preserves the original TME of human tumors together with single-cell readouts that provide granular insights into the mechanism of action of immunotherapy perturbations. GITR agonist had a limited response restricted to cytotoxic CD8 T cells. TIGIT antagonist led to a multicellular transcriptional reprogramming of the TME. This included activation of cytotoxic and dysfunctional CD8 T cells, TFh-like cells, and DCs together with a reduction in the immunosuppressive Treg phenotype.

### Supplementary Information

**Additional file 1.** Supplementary tables S1 – 13.

**Additional file 2.** Supplementary information - supplementary figure legends and supplementary figures S1 – S4.

## Data Availability

The tumor-infiltrating immune cell atlas dataset analyzed in this study is available in the Zenodo data repository at https://zenodo.org/records/4263972 [28]. The gastric cancer scRNA-seq dataset analyzed in this study is available at https://dna-discovery.stanford.edu/research/datasets/ [86]. Sequencing data generated in this study is deposited in NCBI’s dbGaP repository under accession phs001818 at https://www.ncbi.nlm.nih.gov/projects/gap/cgi-bin/study.cgi?study_id=phs001818 [87]. Cell Ranger matrices generated in this study are available on https://dna-discovery.stanford.edu/research/datasets/ [88].

## Abbreviations

TME: Tumor microenvironment
TSCs: Tumor slice cultures
scRNA-seq: Single-cell RNA sequencing
scTCR-seq: Single-cell TCR sequencing
GC: Gastric cancer
Tregs: Regulatory T cells
CRC: Colorectal cancer
TFh-like: Follicular helper-like
DCs: Dendritic cells
MAST: Model-based analysis of single-cell transcriptomics
FDR: False discovery rate
mIF: Multiplex immunofluorescence
ARI: Adjusted Rand Index
ctrl: Control
PMAIono: PMA/Ionomycin
T0: Time-point zero
H&E: Hematoxylin and eosin
UMAP: Uniform manifold approximation and projection
NFAT: Nuclear factor of activated T cells
IFN: Interferon
TR: Transcriptional responsive
TNR: Transcriptional non-responsive
RNA-ISH: RNA in situ hybridization
