# Supplementary material for: GITR and TIGIT immunotherapy provokes divergent multicellular responses in the tumor microenvironment of gastrointestinal cancers
Source: Genome Med. 2023 Nov 26;15:100. doi: 10.1186/s13073-023-01259-3 (PMC10680277; doi:10.1186/s13073-023-01259-3)
Supplement: Supplementary file 2 — Additional file 2. Supplementary information - supplementary figure legends and supplementary figures S1 – S4. [file 13073_2023_1259_MOESM2_ESM.pdf]

**SUPPLEMENTARY INFORMATION**

**Title:** GITR and TIGIT immunotherapy provokes divergent multi-cellular responses in the tumor microenvironment of gastrointestinal cancers


**Authors:**

Anuja Sathe[1], Carlos Ayala[2], Xiangqi Bai[1], Susan M. Grimes[1], Byrne Lee[2], Cindy Kin[2], Andrew Shelton[2], George Poultsides[2], Hanlee P. Ji[1]

**Affiliations:**

[1] Division of Oncology, Department of Medicine, Stanford University School of Medicine, Stanford, CA, United States

[2] Division of Surgical Oncology, Department of Surgery, Stanford University, Stanford, CA, United States

**Corresponding author**

Hanlee P. Ji

Email: genomics_ji@stanford.edu

Phone: +1-650-721-1503

Mailing address: CCSR 2245, 269 Campus Drive, Stanford, CA-94305, USA

## SUPPLEMENTARY FIGURE LEGENDS

**Fig. S1.** (A) UMAP representation of tumor epithelial cells following batch-corrected graph-based clustering of all datasets colored by samples. (B) Dot plot of tumor epithelial cells depicting average expression levels of specific lineage-based marker genes together with the percentage of cells expressing the marker. (C-E) UMAP representation of (C) stromal cells, (D) myeloid cells with inset depicting expression of respective macrophage marker genes and (E) lymphocytes following batch-corrected graph-based clustering of all datasets colored by cell types. (F) Dot plot of B and plasma cells depicting average expression levels of specific lineage-based marker genes together with the percentage of cells expressing the marker. (G) Violin plots depicting the expression of respective genes in CD8 dysfunctional and CD8 dysfunctional proliferating cells.

**Fig. S2.** (A-D) Quantification of mIF across all samples and regions of interests. Error bars indicate standard error of mean. (E-F) Scaled average expression of respective genes in various cell types from each tumor type in the publicly available tumor immune cell atlas dataset. (G) Representative image from H&E staining of a 24 hour ctrl TSC from sample CRC_5. Scale bar = 100 μm

**Fig. S3.** (A) Representative images of RNA-ISH staining for *GZMB* and nuclear stain DAPI in control or TIGIT treated TSCs from samples CRC_5 and GC_1_3. Scale bar = 20 μm. (B) Quantification of *GZMB* RNA-ISH from three representative regions of interest from control or TIGIT treated TSCs from respective samples with t-test *p*. (C) Pathway activity in control and treated CD8 T cells corresponding to baseline non-expanded TCR clonotypes with t-test *p*. (D)

TCR expansion index from T0, ctrl and TIGIT treated CD8 T cells per sample with ANOVA Tukey's HSD *p*.

**Fig. S4.** (A-B) Scaled average expression of respective genes in (A) cytotoxic and (B) dysfunctional CD8 T cells following ctrl, PMA/Ionomycin, GITR or TIGIT treatment. (C-D) Pathway activity with t-test *p* in control and treated TFh-like cells corresponding to baseline (C) expanded and (D) non-expanded TCR clonotypes from GC_1_3 tumor.

Fig. S1

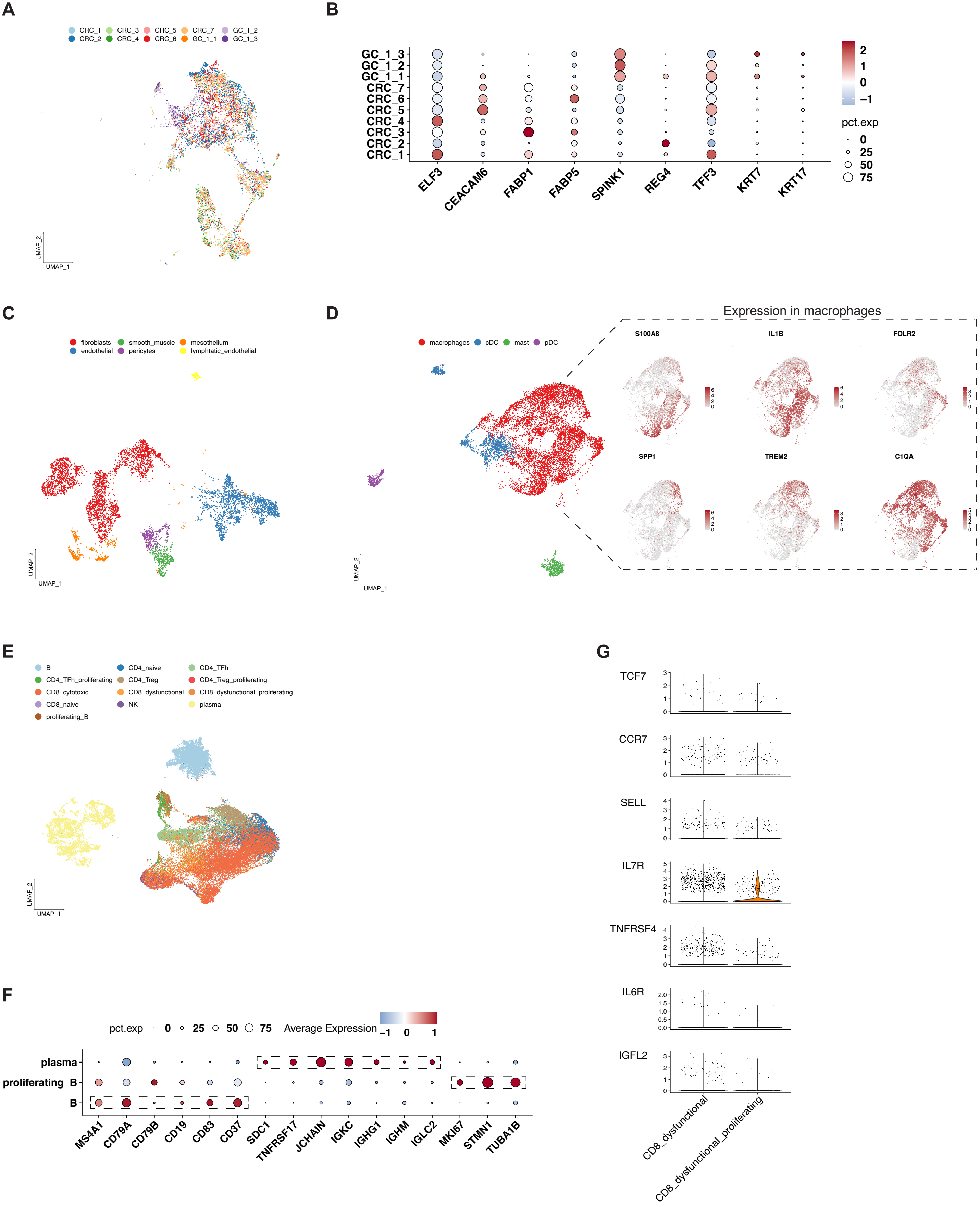
A
CRC_1 CRC_2 CRC_3 CRC_4 CRC_5 CRC_6 CRC_7 GC_1_1 GC_1_2 GC_1_3
UMAP_1
UMAP_2
B
GC_1_3
GC_1_2
GC_1_1
CRC_7
CRC_6
CRC_5
CRC_4
CRC_3
CRC_2
CRC_1
ELF3 CEACAM6 FABP1 FABP5 SPINK1 REG4 TFF3 KRT7 KRT17
pct.exp
0 25 50 75
C
fibroblasts smooth_muscle mesothelium endothelial pericytes lymphtatic_endothelial
D
macrophages cDC mast pDC
Expression in macrophages
S100A8 IL1B FOLR2 SPP1 TREM2 C1QA
E
B CD4_naive CD4_TFh CD4_TFh_proliferating CD4_Treg CD4_Treg_proliferating CD8_cytotoxic CD8_dysfunctional CD8_dysfunctional_proliferating CD8_naive NK plasma proliferating_B
F
pct.exp 0 25 50 75 Average Expression −1 0 1
plasma
proliferating_B
B
MS4A1 CD79A CD79B CD19 CD83 CD37 SDC1 TNFRSF17 JCHAIN IGKC IGHG1 IGHM IGLC2 MKI67 STMN1 TUBA1B
G
TCF7
CCR7
SELL
IL7R
TNFRSF4
IL6R
IGFL2
CD8_dysfunctional
CD8_dysfunctional_proliferating

**Fig. S2**

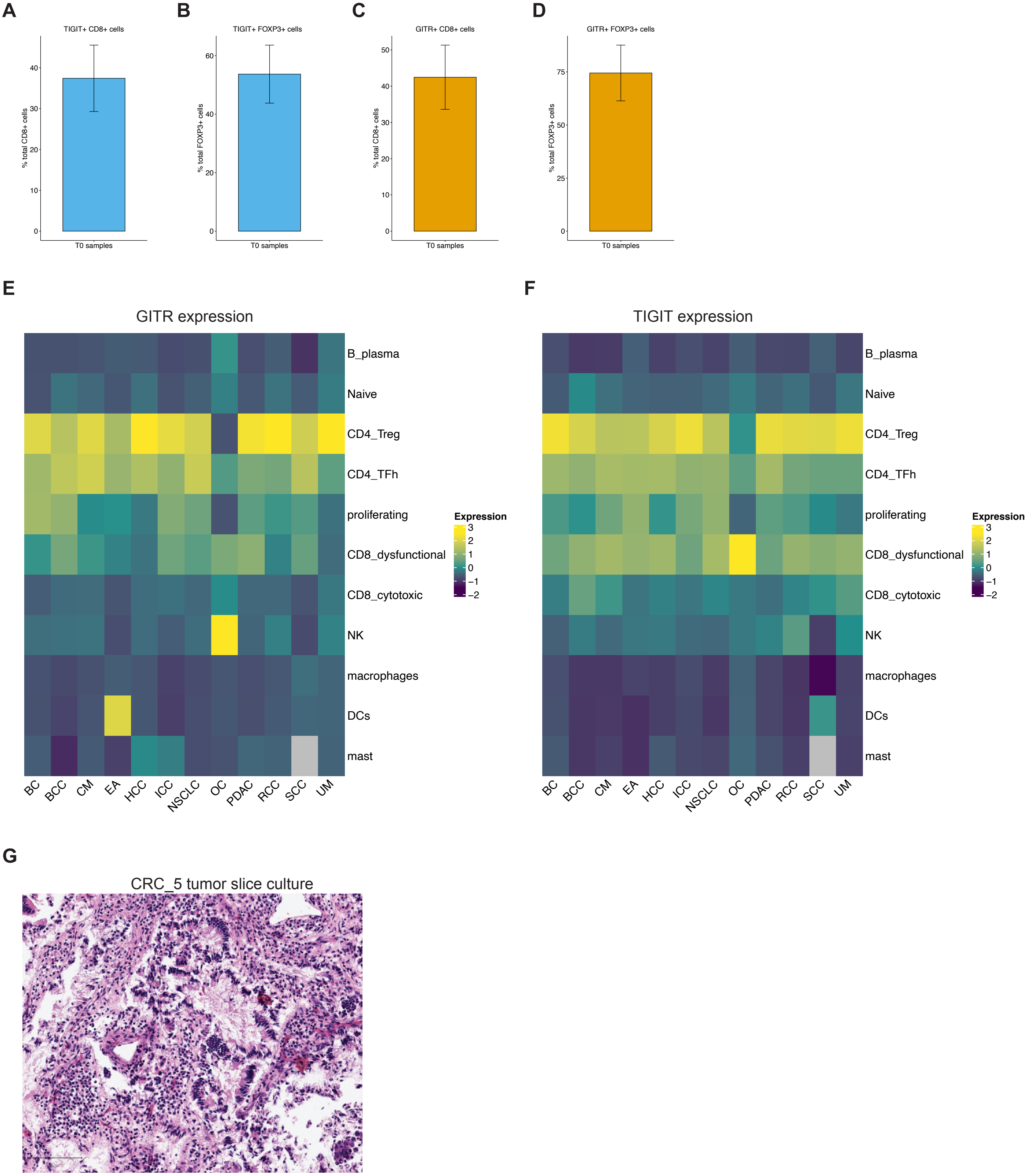
A
TIGIT+ CD8+ cells
% total CD8+ cells
0
10
20
30
40
T0 samples
B
TIGIT+ FOXP3+ cells
% total FOXP3+ cells
0
20
40
60
T0 samples
C
GITR+ CD8+ cells
% total CD8+ cells
0
10
20
30
40
50
T0 samples
D
GITR+ FOXP3+ cells
% total FOXP3+ cells
0
25
50
75
T0 samples
E
GITR expression
F
TIGIT expression
B_plasma
Naive
CD4_Treg
CD4_TFh
proliferating
CD8_dysfunctional
CD8_cytotoxic
NK
macrophages
DCs
mast
Expression
3
2
1
0
−1
−2
BC
BCC
CM
EA
HCC
ICC
NSCLC
OC
PDAC
RCC
SCC
UM
G
CRC_5 tumor slice culture

Fig. S3

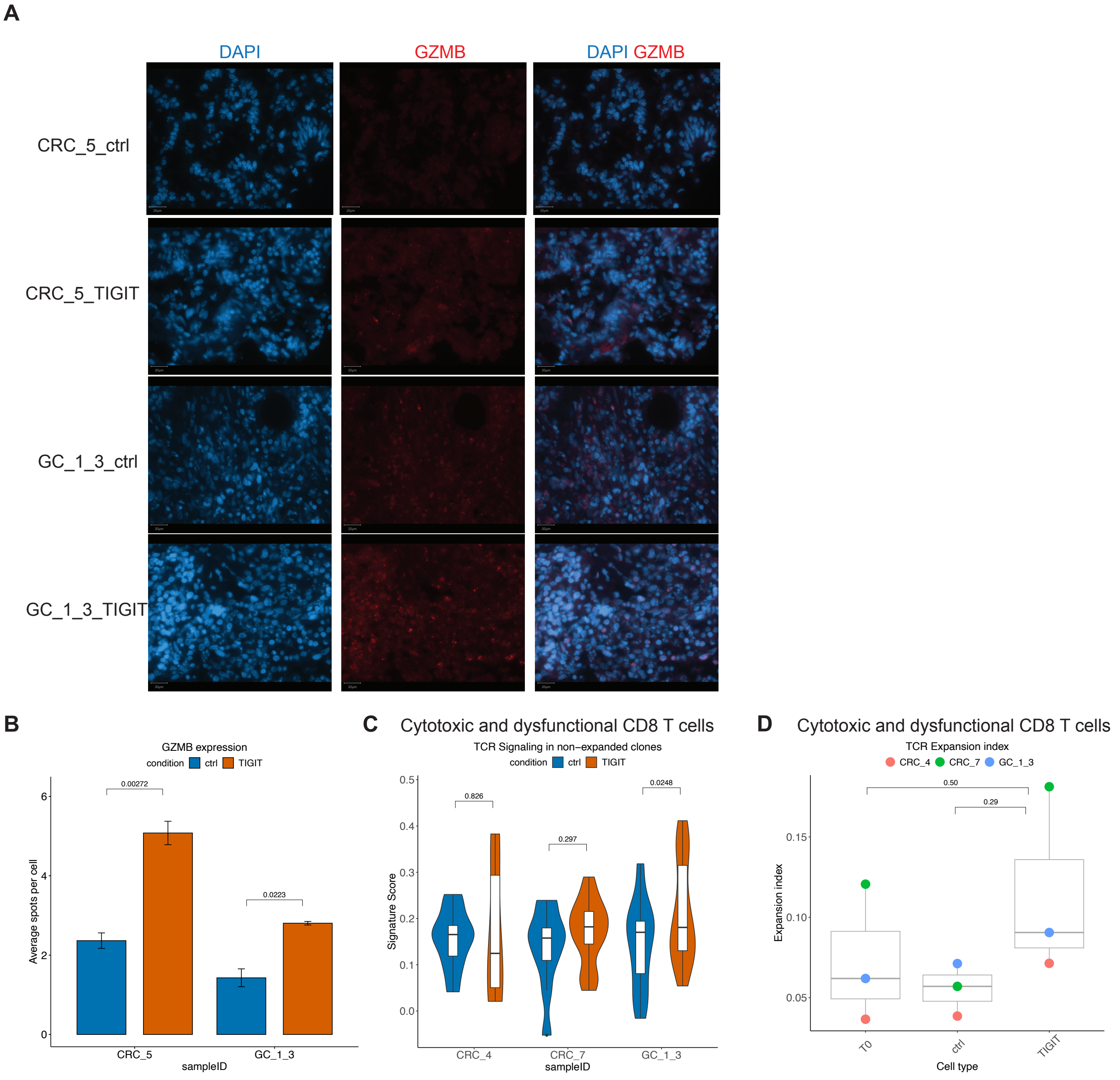
A
DAPI
GZMB
DAPI GZMB
CRC_5_ctrl
CRC_5_TIGIT
GC_1_3_ctrl
GC_1_3_TIGIT
B
GZMB expression
condition ctrl TIGIT
0.00272
0.0223
Average spots per cell
CRC_5
GC_1_3
sampleID
C
Cytotoxic and dysfunctional CD8 T cells
TCR Signaling in non−expanded clones
condition ctrl TIGIT
0.826
0.297
0.0248
Signature Score
CRC_4
CRC_7
GC_1_3
sampleID
D
Cytotoxic and dysfunctional CD8 T cells
TCR Expansion index
CRC_4 CRC_7 GC_1_3
0.50
0.29
Expansion index
T0
ctrl
TIGIT
Cell type

**Fig. S4**

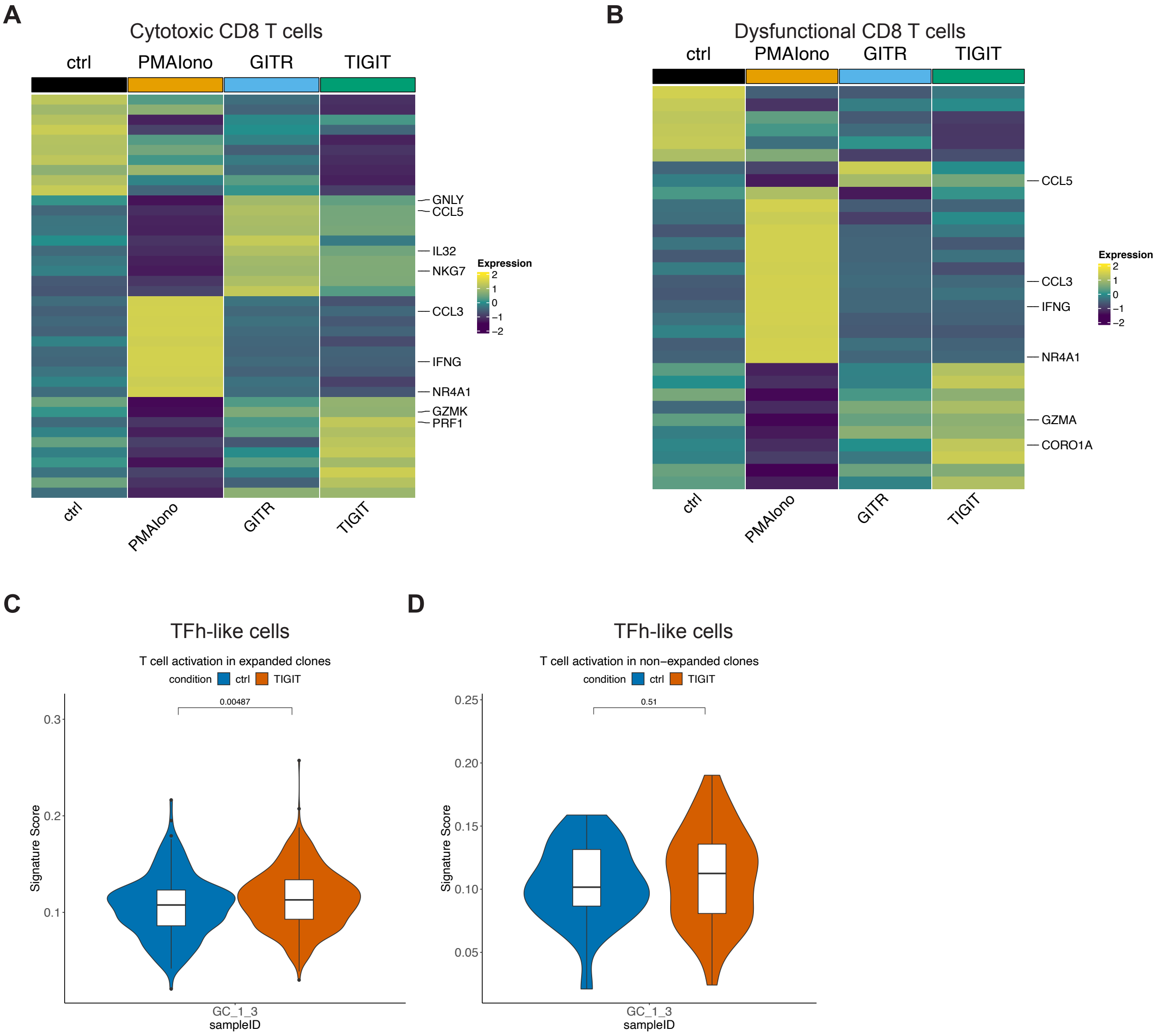
A
Cytotoxic CD8 T cells
ctrl
PMAIono
GITR
TIGIT
GNLY
CCL5
IL32
NKG7
CCL3
IFNG
NR4A1
GZMK
PRF1
Expression
2
1
0
−1
−2
B
Dysfunctional CD8 T cells
CCL5
CCL3
IFNG
NR4A1
GZMA
CORO1A
C
TFh-like cells
T cell activation in expanded clones
condition
0.00487
Signature Score
GC_1_3
sampleID
D
T cell activation in non−expanded clones
0.51
